# Flipping food during grilling tasks, a dataset of utensils kinematics and dynamics, food pose and subject gaze

**DOI:** 10.1038/s41597-021-01101-8

**Published:** 2022-01-12

**Authors:** Débora Pereira, Yuri De Pra, Emidio Tiberi, Vito Monaco, Paolo Dario, Gastone Ciuti

**Affiliations:** 1 The BioRobotics Institute, Scuola Superiore Sant’Anna, Pisa, 56127 Italy; 2 Department of Excellence in Robotics & AI, Scuola Superiore Sant’Anna, Pisa, 56127 Italy; 3 The Research Hub by Electrolux Professional SpA, AD&T, Pordenone, 33170 Italy; 4 University of Udine, Department of Computer Science, Mathematics and Physics, Udine, 33100 Italy

**Keywords:** Research data, Biomedical engineering

## Abstract

This paper presents a multivariate dataset of 2866 food flipping movements, performed by 4 chefs and 5 home cooks, with different grilled food and two utensils (spatula and tweezers). The 3D trajectories of strategic points in the utensils were tracked using optoelectronic motion capture. The pinching force of the tweezers, the bending force and torsion torque of the spatula were also recorded, as well as videos and the subject gaze. These data were collected using a custom experimental setup that allowed the execution of flipping movements with freshly cooked food, without having the sensors near the dangerous cooking area. Complementary, the 2D position of food was computed from the videos. The action of flipping food is, indeed, gaining the attention of both researchers and manufacturers of foodservice technology. The reported dataset contains valuable measurements (1) to characterize and model flipping movements as performed by humans, (2) to develop bio-inspired methods to control a cooking robot, or (3) to study new algorithms for human actions recognition.

## Abstract (summary)

**Table:** 

Measurement(s)	Video Recording • bending force • pinching force • torsion torque • ambient temperature • gaze and pupil positions • 2D trajectory of food • 3D trajectory of utensils
Technology Type(s)	digital camera • strain gauge • Thermocouple Device • eye tracking device • optoelectronic motion capture • convolutional neural network
Factor Type(s)	food type • utensil
Sample Characteristic - Organism	Homo sapiens
Sample Characteristic - Environment	indoor kitchen

Machine-accessible metadata file describing the reported data: 10.6084/m9.figshare.16836670

## Background & Summary

Flipping food, typically when cooking, is not straightforward, either for humans^1^ or robots^2^. This movement is applied to different food (*e.g*., meat, vegetables, bread, eggs)^2–4^ with heterogeneous characteristics (*e.g*., greasy, adherent, deformable) that influence the movement stability, duration and outcome^1,2^.

This is still a poorly understood and rarely automated action^5^: past studies exclusively addressed hamburgers^2^ or pancakes^4^ flipping; state-of-the-art methods to automate this movement have a limited robustness to different and uncertain levels of stickiness and slipperiness^2^. Yet, the strategies of professional, expert cooks were never investigated or modeled, *e.g*., with the motion primitives’ paradigm^6,7^. Specifically, kinematic and kinetic data would help characterizing their strategies^8^.

Such data are, in fact, useful for various scientific fields including cooking education^3^, automation and robotics science^2,4^, or human activity science^9–11^. Furthermore, foodservice machinery^5,12,13^, home appliances^14^ or food manufacturing^15^ industries also have interest in these data to develop autonomous/automatic systems to flip food.

This article presents a dataset for such aims. The 3D trajectories of a spatula and tweezers were acquired during 2866 flipping movements, performed by chefs and home cooks, with grilled hamburgers, chicken breast, zucchini and eggplant slices. The pinching force of the tweezers, the bending force and torsion torque of the spatula were also recorded, as well as RGB videos and the subject gaze. Forces and torques were already used in^1^ for a preliminary analysis showing that bending forces on the impact and maximum pinching forces are adjusted to the food by both chefs and home cooks.

Indeed, this is a novel dataset: systematically searching Scopus and Web of Science reveals very few datasets including food flipping movements^16–18^. They include varied tasks, but each one recorded in a single condition (*e.g*., always with the same food). In^16^, subjects flipped an omelet (among other tasks), but by turning the pan, covered with a lid, instead of using a spatula. In^17^, two recorded actions (both with a spatula) are somewhat related to food flipping: “scrape a piece of dough from a table” and “flip bread on a pan”. The former resembles the first elementary movement that some subjects execute when flipping meat, *i.e*., scrapping (to unstick the meat from the surface). The latter is a type of flipping action without the influence of stickiness. This condition is also present in our experiments, but with vegetables. Like ours, their dataset contains position/orientation and force/torque signals. The third dataset^18^ includes an action of “turning pancakes” but, once more, without a utensil: throwing the pancake with the pan. The three datasets also differ from ours on the context in which the task was performed. In our setup, the subject flipped food without any support from surrounding obstacles, over a plane surface, and the action was constrained only by other food pieces lying beside it. In the other datasets, the task was performed in a pan, so the subject movement can be supported or disturbed by the pan walls.

Moreover, most published experiments use inorganic food models for the study of cooking tasks, *e.g*. cardboard/plastic pancakes^18,19^, polystyrene cakes or even empty eggshells^16^. However, these are incomplete representations of real cooked food, because they lack the important ability of releasing fluids (water, fat) and hardly reproduce the deformability of food and the stickiness of its compounds (*e.g*. proteins). Instead, our dataset was collected in a setup tailored to achieve a compromise between the realism of freshly cooked food (and, thus, the naturalness of movements) and the exposure of sensors to high temperature, fluids and dirty that could damage them or disturb their measurements.

Finally, additional data were derived offline to complement the acquired dataset: 2D trajectories of the food, estimated from the videos, using a custom Computer Vision method. These data were intended to support the analysis of kinematic/kinetic data. Yet, both the data and the method may also be useful for machine/deep-learning researchers to benchmark different food detection algorithms. Furthermore, our method could be implemented in a vision system, for real-time food monitoring or guidance of a robotic manipulator (*e.g*., for the foodservice industry).

The scientific community may also find our dataset useful to model flipping movements not only for robotic applications but also to study methods of human actions recognition^9–11^.

## Methods

To what concerns the acquisition of forces and torques and the general acquisition protocol, the methods described below include a detailed and expanded version of the description stated in our related work^1^.

### Study participants

Recruitment in the study was extended only to subjects affiliated to Electrolux Professional SpA, for a matter of confidentiality of all the exposed equipment and products in the private laboratories of the company. A communication of the study occurrence and invitation to participate was expressed in person and also by e-mail. Professional cooks of the Chef Academy of Electrolux Professional SpA (EP) and ordinary home cooks (*i.e*. not working/having worked as a professional cook) were recruited for the study and allocated in two groups, *chefs* and *not-chefs*, respectively. Four *chefs* (all male, with 24 ± 11 years of job experience in foodservice) and five *not-chefs* (2 female and 3 male, only with experience in cooking at home) volunteered to participate. All subjects were healthy individuals without any known skeletal and/or neuromuscular disorder or any injury that could hamper or affect the task performance.

Before participating in the experiments, all subjects were given an oral and written explanation of the experiments and they gave their written informed consent. The experiments were conducted in accordance with the World Medical Association Declaration of Helsinki on Ethical Principles for Medical Research Involving Human Subjects and were approved by the R&D Director and the Human Resources Director of Electrolux Professional SpA, as responsible delegates for the safety and ethics review of research activities in the company. The subjects, after giving their consent, were asked some personal information to support the data analysis, *i.e*. years of professional experience in foodservice, age, gender, hand dominance, weight, height, arm length. These data are presented in Table 1. Some of these are in the form of ranges to respect the participants privacy.

**Table 1:** Basic demographics and body measurements of the participants.

Participant	S1	S2	S3	S4	S5	S6	S7	S8	S9
Group	chef	chef	chef	notchef	notchef	notchef	notchef	notchef	chef
Experience in foodservice (years)	25	15	30	0	0	0	0	0	17
Age range (years)	51–60	31–40	41–50	21–30	21–30	21–30	21–30	31–40	31–40
Gender	male	male	male	female	female	male	male	male	male
Hand dominance	right	right	right	left	right	right	right	right	right
Weight range (kg)	71–90	71–90	>110	51–70	51–70	51–70	51–70	91–110	71–90
Height range (cm)	161–170	161–170	181–190	171–180	161–170	171–180	171–180	181–190	171–180
Arm length (cm)	52	50	64	55	52	52	58	55	50

The experience in foodservice accounts for any job that the participant had in restaurants, canteens, bars, buffets, cafeterias, catering services agencies, and other foodservice contractors, following the definition of foodservice according to the North American Industry Classification System^54^, commonly used in the hospitality industry^55^.

### Data acquisition setup

**Fig. 1:**
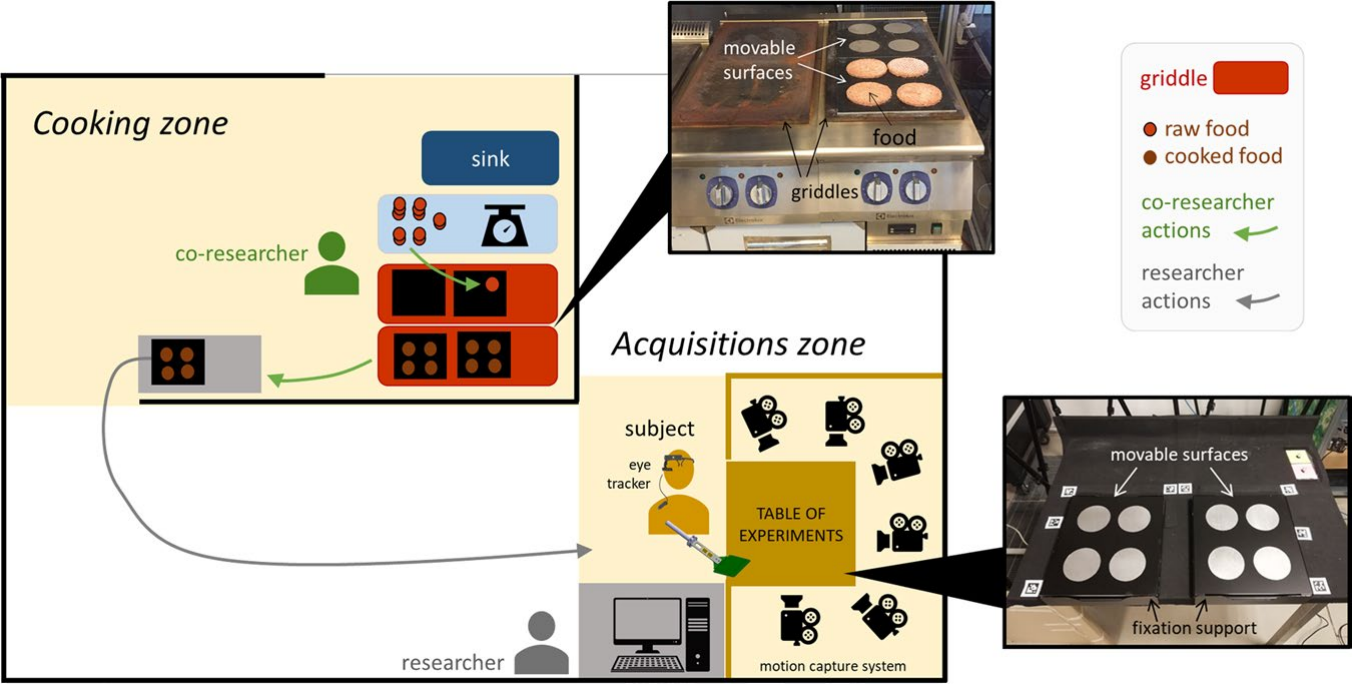
Experimental setup in the laboratory.

All trials were conducted in the private facilities of Electrolux Professional SpA, in Pordenone (Italy), in the I-lab of the Advanced Development & Technologies Department. A custom experimental setup, illustrated in Fig. 1, was created in the I-lab having two stations in separated parts of the laboratory: one for cooking (where there was a professional griddle, under a hood) and another for the acquisitions (where the subject was located, as well as a table and all the equipment for the acquisitions).

#### Motion capture system

A 6-camera optoelectronic system (Vicon Motion Systems Ltd., Oxford, UK) and the proprietary software (Vicon Nexus, version 2.7.0, Oxford Metrics Ltd., UK) were used to track the 3D trajectories of a set of passive infrared-reflective (IR) markers (diameter of 14 mm) attached to the utensils and on the experiments table. This motion capture (MoCap) system was composed of five Vicon Bonita cameras and one Vicon Vero camera. The configuration of the cameras was set around the experiments table so that each IR marker was visible, at least, to three cameras. The sampling frequency was set to 100 Hz.

The surfaces, that were used to present the food to the subject, were painted black to reduce undesirable light reflections caused by the polished metal and, thus, to avoid disturbing the acquisitions with the MoCap system. The ink was just not placed on designated parts of the surface for the food placement, to avoid interfering with the natural adhesion of food to the metallic surface and also to mark the position of the food (for repeatability among trials).

**Fig. 2:**
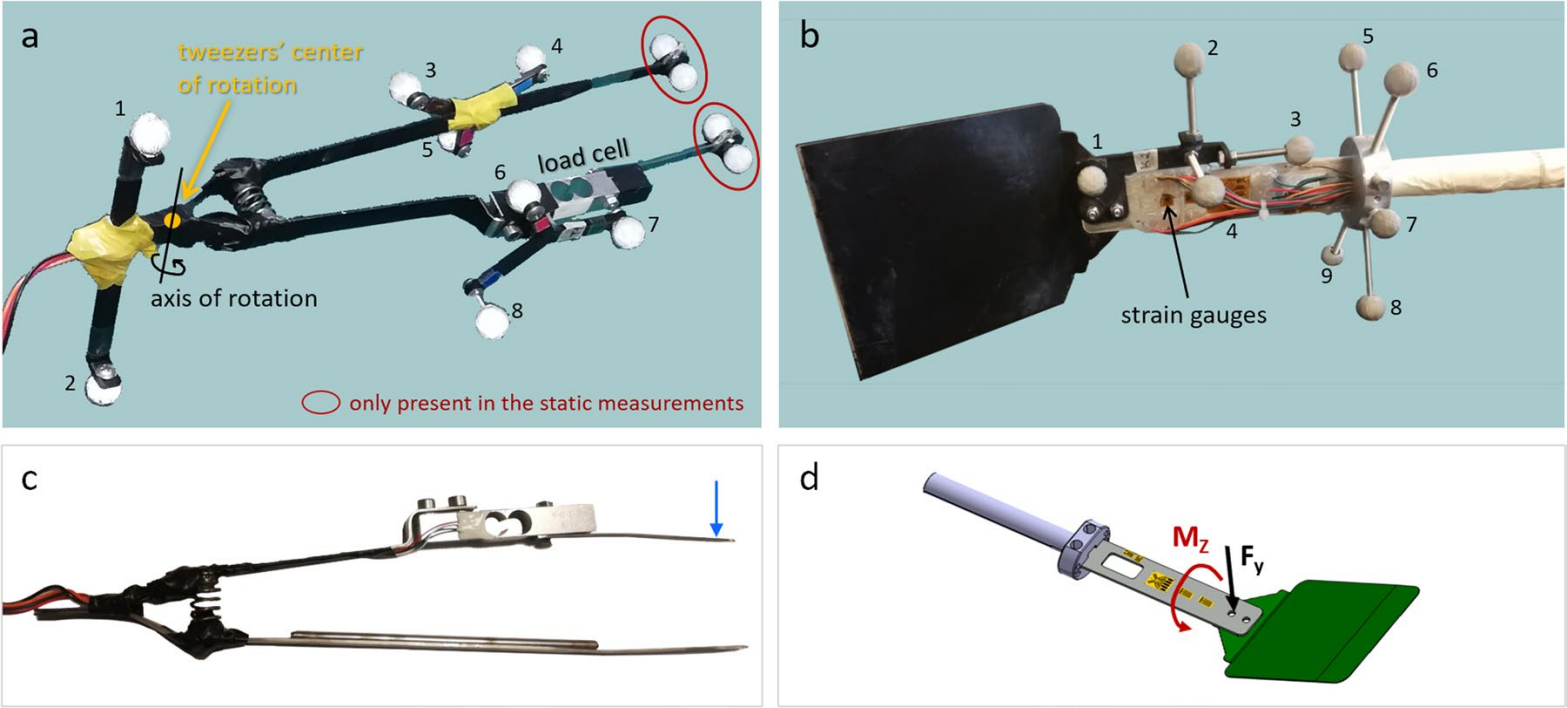
Instrumented kitchen-utensils with markers for motion capture (MoCap) and transducers for force and torque measurements: (**a**) tweezers markerset; (**b**) spatula markerset; (**c**) positive sense of the pinching force; (**d**) positive sense of the bending force (*F*_*y*_) and torsion torque (*M*_*z*_). The numbers in **a** and **b** identify the MoCap markers labelled in the dataset. The markers in red were not present in the experiments, but only in the static measurements performed for the calculation of the position of the tweezers tips.
- The spatula can be approximated to a system of two rigid links (*i.e*. the spatula handle and the spatula blade) connected by a bendable beam. Hence, it was convenient to track the motion of both links. A cluster of markers was, then, attached to the handle and another one to the spatula blade, as shown in Fig. 2b.
  Two IR markers were attached to the table of experiments as references to support the data labelling and also to be tracked in the videos with the algorithm described in “Video processing to track food”.
  In each day of experiments, the cameras were calibrated, following the manufacturer instructions^20^ and the standard practise in calibrating optoelectronic systems^21–23^. Calibration was done always before the trials and after any contingent event (*e.g*., pauses or bumped cameras), even if the latter rarely occurred. The calibration was considered successful if the errors obtained from the calibration procedure (indicated by the software as “World Error”, for each camera) was below 0.1 mm^24^. Otherwise, the calibration would be repeated. Before any dynamic trial, short static trials (*i.e*., with the object still on the table) were recorded. These trials were performed for:
- the markers on the tweezers (to create the model template in Nexus);
- the markers on the spatula (to create the model template in Nexus);
- several markers placed on the left and right surfaces, so that, if desired, one can determine the plane defining these surfaces - this may be useful for the calculation of relative spatio-temporal features, such as the orientation of the utensil with respect to the table;
- three markers aligned on a support in the direction of the gravity vector, which may be useful for researchers studying the forces and torques during flipping movements.

A custom markerset was defined (and unchanged for all the trials and subjects) to allow the subsequent reconstruction of the utensils assuming the following kinematic models:

- Kitchen-tweezers can be approximated to a system of two rigid links moving around a joint, with a spring between them (such that, if a force is applied to open the tweezers and then removed, the links return to the rest position, always with the same distance between them). Therefore, the motion of each link of the tweezers needed to be captured, so, a cluster of IR markers was attached to each link. Although three markers are enough to obtain the frame (*i.e*. the coordinate system) of each link over time, it is convenient to have, at least, one extra marker to compensate for eventual missing data during the acquisitions. It would be difficult, however, to place more than three markers on each link because, during the flipping movement, the tweezers get very close to the table and the markers could touch the table and disturb the subject’s movements. Thus, only three markers were attached to the tweezers links and an additional pair of markers was placed on the extremity of the tweezers where the subject grasps this utensil (see Fig. 2a).

An additional static trial was performed once, with extra markers on the tweezers tips (as shown in Fig. 2, in red), to accurately measure the distances between all the markers used for the dynamic trials and the tweezers tips. This way, the position of the tips could be estimated in the dynamic trials, offline, as explained in “MoCap data labelling and pre-processing”. Similarly, IR markers were placed in three corners of the spatula blade (in addition to the markers used in the experiments) and a static trial was recorded, so that the spatula plane could later be obtained for the dynamic trials. Naturally, none of these extra markers could be placed on the utensils during the dynamic trials as they would disturb the subject’s task.

**Fig. 3:**
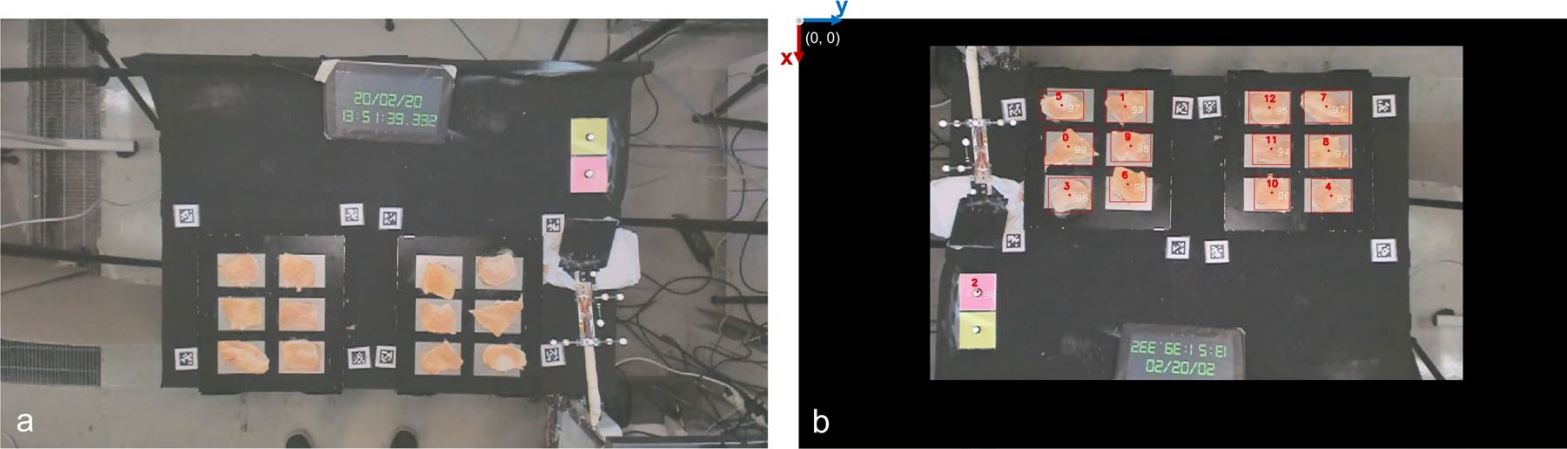
The table of experiments. (**a**) A frame from the original videos of the experiments (after a proper rotation to turn the clock numbers to the reader’s view). (**b**) The same frame (with the original orientation of the camera), taken from the output video produced by the food tracking algorithm: the black region around the image is the part that was darkened, around the experiments table (ROI); the red numbers are attributed by the algorithm to enumerate the objects found in the image (and correspond to the object ID in the CSV files); these numbers are placed on the object’s centroid; the white numbers near the objects are the precision of the object class identification; in the top-left corner there is the reference frame, used for the centroids’ position.

The distances between markers in the same segment of the utensils are listed in the Supplementary Table 1. In Supplementary Fig. 2 and Fig. 3, there is a scheme with all the relevant dimensions of the tweezers and the spatula, respectively.

The names used to label markers are listed in Table 2.

**Table 2:** List of labels identifying the IR markers used for motion capture in the experiments.

Marker label	Description	Number in Fig. 2 (panel a or b)
sFar	spatula blade, the most distant marker from the subject	1 (b)
sR	spatula blade, marker at right	2 (b)
sClose	spatula blade, the closest marker to the subject	3 (b)
sUp	spatula blade, the upper-most marker	4 (b)
hDownR	spatula handle, the down-right marker	5 (b)
hUpR	spatula handle, the up-right marker	6 (b)
hMid	spatula handle, the up marker in the middle	7 (b)
hUpL	spatula handle, the up-left marker	8 (b)
hDownL	spatula handle, the down-left marker	9 (b)
c_tipother	one of the markers on the grasping extremity of the tweezers	1 (a)
c_tiplong	one of the markers on the grasping extremity of the tweezers	2 (a)
a_long	tweezers arm without load cell, the side marker with a long stick	3 (a)
a_far	tweezers arm without load cell, the most distant marker from the subject	4 (a)
a_short	tweezers arm without load cell, the side marker with a short stick	5 (a)
c_short	tweezers arm with load cell, the side marker with a short stick	6 (a)
c_far	tweezers arm with load cell, the most distant marker from the subject	7 (a)
c_long	tweezers arm with load cell, the side marker with a long stick	8 (a)
logRefp	reference marker for the videos, over the pink square on the table	—
logRefy	reference marker for the videos, over the yellow square on the table	—
gUp	the upper-most marker used for the gravity vector	—
gMid	the middle marker used for the gravity vector	—
gDown	the down marker used for the gravity vector	—

#### Force/torque sensors

Two utensils, introduced in^1^, were instrumented to measure forces and torques of interest (see Fig. 2). Common kitchen-tweezers with long and thin tips (1 mm thick, 320 mm long and 3 mm wide) were modified to integrate a commercial uniaxial load cell. The pinching force (between ±10 N) was, this way, measured during the experiments. A spatula was designed within typical dimensions: blade of 117 mm × 110 mm, 1.5 mm thick, sharp edge; blade tilting angle of 145°; handle with 25 mm of total length, and 19.6 mm of diameter in the grasping segment. A custom handle was built having a bendable beam (1.5 mm thick, 30 mm wide, 140 mm long), where strain gauges were placed and rigidly fixed for the measurement of compression and bending forces, as well as torsion torques. As discussed in^1^, the calibration for the compression forces could not be used, thus, compression forces are not included in this dataset. Additionally, two welded-tip K-type thermocouples were attached to the beam of the spatula handle (one in the bottom face, another in the top face) to monitor the temperature variations near the strain gauges. For more details on the construction and validation of these instruments, we invite researchers to read^1^.

An industrial-grade PLC (model X20CP3584, B&R Industrial Automation GmbH, Austria) was used to acquire the forces/torques and temperature signals. To collect data, proprietary acquisition hardware modules were connected to the PLC, *i.e*. the X20AIB744 module, to read signals from the load cell in the tweezers and the strain gauges arranged in Wheatstone bridges in the spatula; the X20AT402 module, to read the thermocouples signals. The signals were acquired with Simulink^TM^ (version 10.0, Mathworks Inc., USA) and the software from the PLC manufacturer, *i.e*. Automation Studio (AS), that were integrated through the AS Target for Simulink. In this software, the acquisition modules were configured and the sampling frequency selected to 100 Hz. The Wheatstone bridges were supplied with 5.5 VDC, and the analog signals digitally converted with 24 bits of resolution. In the dataset, we provide both the original raw data and the forces and torques (in Newton and Newton.metro, respectively) converted from the raw data using the instruments calibration curves (Eq. 1) and the Eq. 2. The direction of positive forces is indicated in Fig. 2, panels c and d.

1 $$\begin{array}{lll}{F}_{pinching} & = & 3.03\cdot 1{0}^{-4}\cdot rawData-1.19\\ {M}_{torsion} & = & 1.6967\cdot 1{0}^{-05}\cdot rawData-0.0265\\ {M}_{bending}^{A} & = & 1.1151\cdot 1{0}^{-05}\cdot rawData+0.44916\\ {M}_{bending}^{B} & = & 1.0733\cdot 1{0}^{-05}\cdot rawData-0.014288\end{array}$$

2 $${F}_{bending}=\frac{{M}_{bending}^{A}+{M}_{bending}^{B}}{{l}_{AB}}$$

*rawData* are the raw measurements from the respective Wheatstone bridge in the spatula or the tweezers. The distance between bridges A and B, in the spatula, *l*_*AB*_, is 13 mm (from the midline of one strain gauge to the midline of the other).

#### Digital camera

The experiments were filmed from above the table of experiments, using a Logitech Brio video camera (Logitech Europe S.A., Lausanne, Switzerland), positioned parallel to the table. An acquisition frequency of 90 fps was selected and an image resolution of 720p (HD). A set of white LEDs were used to illuminate the table for a suitable image quality both with this digital camera and the eye tracker. The CL-Eye test software was used to capture the video because it allows us to customize several parameters of the image acquisition in a friendly and easy-to-use app. We were particularly interested in controlling the exposition time because the value of this parameter cannot be too high (*i.e*. too long exposition time), otherwise, one cannot achieve 90 fps. The camera was connected through a USB 3.0 port.

#### Eye tracker

The subjects wore a wearable eye tracking headset by Pupil Labs GmbH (Berlin, Germany), with a monocular eye camera mounted on the right eye. This is a low-cost but high-quality headset that has two types of cameras: one that records the subject’s field of vision (the world camera) and a second one that records the subject’s eye movements (the eye camera). The eye gaze was recorded using the Pupil Capture app (version 1.21.5) provided by the manufacturer, which includes their algorithms to automatically detect the participant’s pupil and to correlate the world images with the eye images. The world video was captured at 30 fps with a resolution of 1280 × 720, and the eye video was captured at 120 fps with a resolution of 320 × 240. The remaining parameters were left to their default value in recording mode.

Before starting every experimental session, the headset was placed on the subject’s head and the calibration procedure (with the screen marker method) was performed, following the instructions from the manufacturer, found in their documentation. The authors assessed the outcome of each calibration by checking, at the end, the residual between reference points and matching gaze positions that were recorded during calibration. This measure is displayed, in degrees, by the Accuracy Visualizer plugin of Pupil Capture (*i.e*., the supplier’s software). The authors considered a maximum acceptable threshold for this residual of 2 degrees, similarly to other researchers, e.g.^25^. When it was exceeded, the authors tried to re-adjust the position of the eye camera to fit better the subject’s eye/eyelashes and the calibration was repeated.

Eight markers from the tag36h11 family of Apriltag (available with Pupil Labs documentation) were printed on paper and covered with transparent adhesive tape for protection against fluids. These markers were placed around the corners of the left and right surface, glued on the experiments table (as observable in Fig. 3), to define areas of interest (surfaces) to be tracked synchronously with the eye gaze.

#### Synchronization

Different signals needed to be acquired by two different computers:

1. the Vicon system was connected to one computer where Nexus was installed;
2. another computer was connected to the remaining sensors: the eye tracker, the digital camera and the PLC reading the force/torque sensors and temperature.

**Fig. 4:**
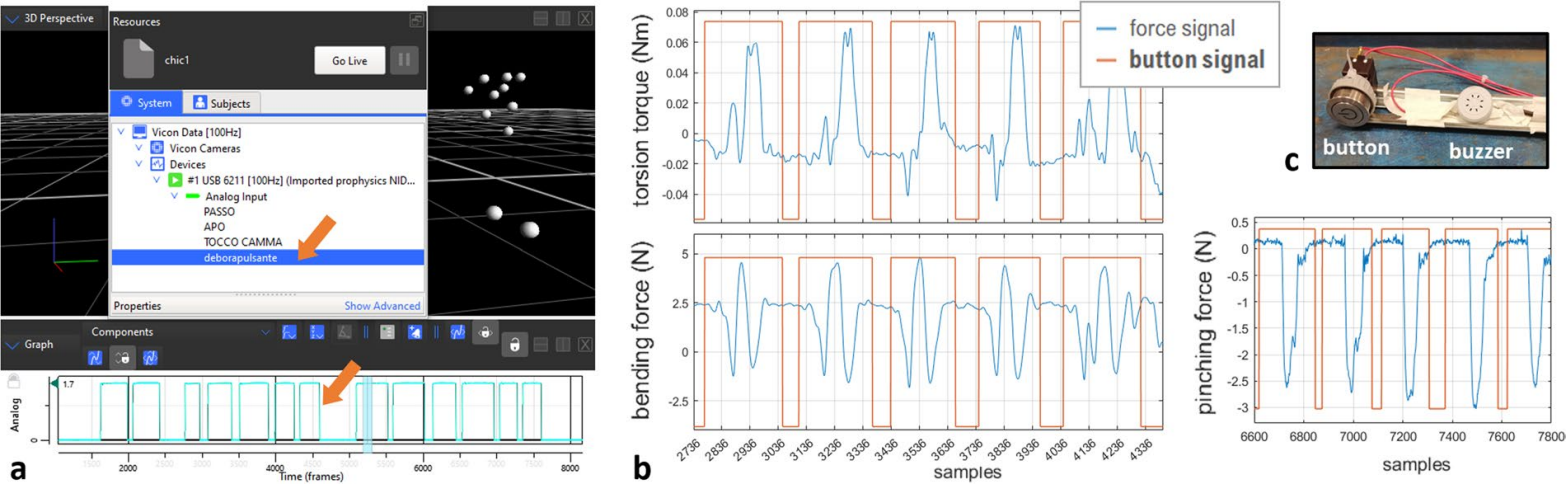
The button signal used for sensors synchronization and control of the experiments flow: (**a**) the button voltage signal integrated in the C3D files (under “System”>“Devices” in Nexus software; highlighted by the orange arrow); (**b**) the button voltage signal integrated in the force data files (examples with forces measured with the spatula and the tweezers); (**c**) the button and the buzzer used in the experiments.

To synchronize the signals, two methods were integrated:

1. *Detecting the start of some of the acquisition programs*:
  An acquisition in Nexus can be started/stopped by pressing “CTRL + ENTER”; in Pupil Capture, an acquisition can be started/stopped by pressing “R”; and the video record in CL-Eye program starts by clicking in the menu option “Start Capture” and, then, pressing “ENTER”. Knowing this, the start of these three data streams was identified by the letter pressed in the keyboard and, this way, video, eye tracking data and MoCap data were synchronized.
  To do so, the open-source LSL (Lab Streaming Layer^26^) was used. LSL is a network for the synchronized collection of sensors signals in research experiments through clock offset measurements^27,28^. The LSL Keyboard app (version 1.10, downloaded from their GitHub page) and the LSL LabRecorder app (version 1.12, by Swartz Center for Computational Neuroscience, downloaded from their GitHub page) were running on each computer independently. The keyboard app detects keyboard presses and associates these events to a data stream in the LSL network. This way, the LabRecorder app “listens to” this stream, assigns a precise timestamp to any detected keyboard press in the stream and saves the timestamps and events in an XDF file.
  Note that, at the beginning of each experimental session, the researchers ran a custom code (see section “Code availability”) that would shortly establish a TCP/IP communication between the two computers (PC), in order to exchange a message and a reply between the PCs. In simultaneous, the LabRecorder app was running and assigning timestamps to the exchanged messages. This way, it was possible to determine the time difference between the clocks of the two PCs and, later, use this difference to synchronize the timestamps of the data collected by the two PCs.
2. *Marking the start of each movement*:

The start of the acquisitions in Simulink could not be tracked by a keyboard press, so another method was necessary – a button was connected to the PLC and its voltage was measured by the acquisition module B&R X20AI4622 and, thus, recorded in Simulink (see Fig. 4b); the same button was connected to a DAQ card (NI USB-6211, National Instruments Corp., Texas, USA), installed in Nexus as an analog device, to also read the button voltage synchronously with the 3D motion (see Fig. 4a); then, during the trials, the button was pressed ON and OFF by the researcher, respectively, before the start and after the end of each movement, as explained in “Protocol description”; this way, by superimposition of the records of the button signal, MoCap data were synchronized with forces/torques.

For redundancy, in case one of the above methods could not be used in some experiment, additional actions were taken:

1. A millisecond-precision clock was displayed by a tablet placed on the experiments table, so that it could be seen by both the eye tracker and the digital camera. The Android app “AtomicClock” was used to obtain the clock.
2. At the beginning of every trial, the utensil was always left static on the table; this way, synchronization can also be attained by detecting the rapid change in the signals caused by the subject when picking up the utensil from the table.

Indeed, this redundancy was useful with subject S9 because, due to unforeseen technical difficulties in this day, both the DAQ card and the tablet with the clock were not available in the laboratory. Therefore, for the trials with S9, synchronization was achieved by detecting the precise instant in which the subject picked up the utensil from the table – an evident event observed in every collected signal. The reader should note that all signals (motion, forces/torques, eye gaze, videos) were collected uniformly among subjects, including S9. The only difference with S9 is in the method of synchronization between the different signals, as explained above. Even if this difference is not expected to cause a significant bias in the data (as one can see in the section “Validation of the synchronization method used with subject S9”), users are still advised to pay special attention to the data of this subject.

### Acquisition protocol

Each subject participated in one experimental session of, approximately, 1.5 h. In addition, before the experiment, 30 minutes were occupied with the initial preparation that consisted in: placement of the eye tracker on the subject and calibration; remind the subject of the experimental protocol/setup and answer any doubts of the subject; 10–15 min for the subject to practice the movements of flipping food and to get familiarized with the setup; initiate cooking the first batch of food pieces.

#### Recorded tasks

The experimental session included several trials for the subject to repeat flipping movements: first with meat, then, with vegetables. A pause of about 10 minutes was made between trials with meat and trials with vegetables for the subject to rest.

**Fig. 5:**
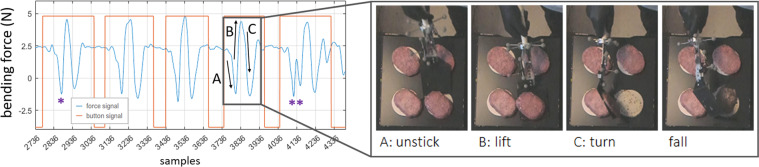
Phases of a food flipping movement. These phases are reflected in the bending force profile, as observable at the left. With vegetables, it is not usually necessary to unstick, but only to pick up. Similar phases are noticed with tweezers. The sign *indicates an example of a movement in which a single stroke was applied to unstick/pick up the food, while ** indicates an example in which two strokes were applied by the subject. Note: this figure is part of the content of Fig. 9 of our related work^1^.

A flipping movement consists of picking up the food and turning it upside down (as depicted in Fig. 5). For food that sticks, picking it up involves unsticking the food from the surface.

**Fig. 6:**
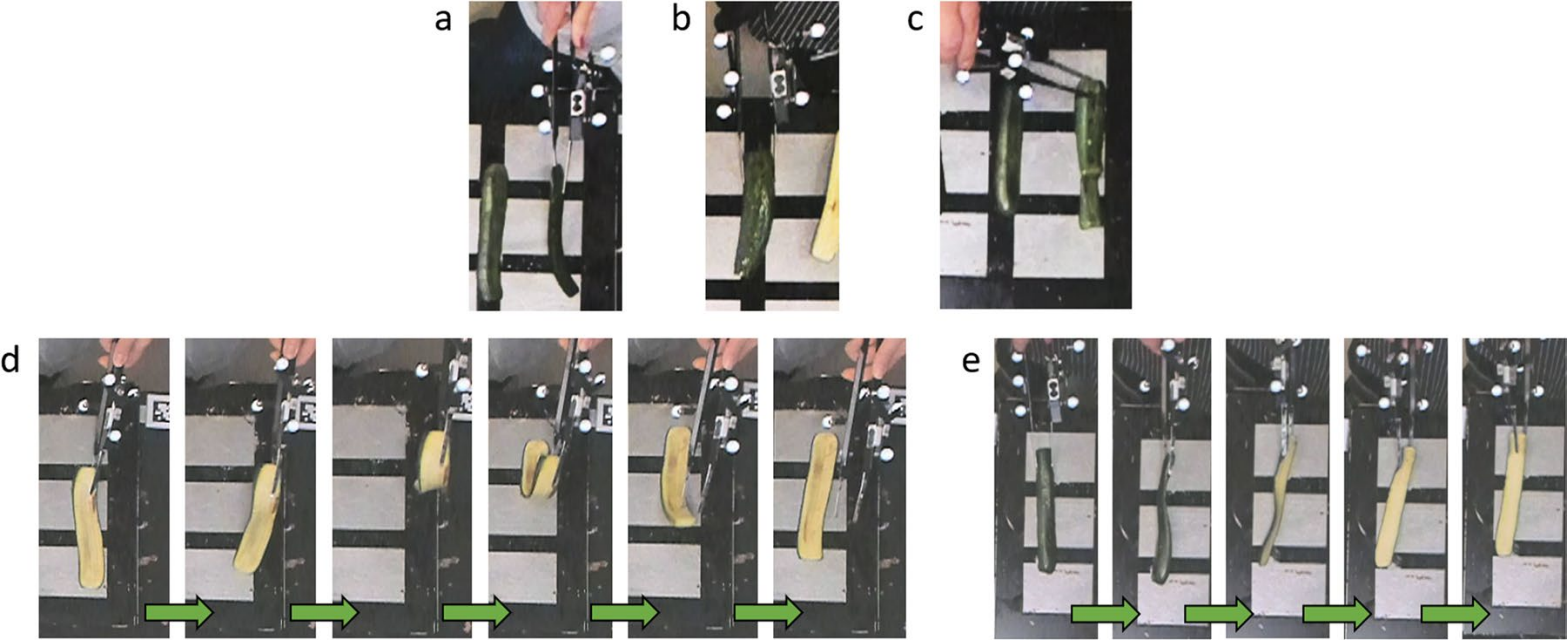
Some strategies selected by the subjects to pick up and to turn the food. Picking and turning are phases of the flipping movement. (**a**–**c**) pickup strategies; (**d**,**e**) turning strategies.

The flipping action can be classified as a non-prehensile object manipulation problem^29^. No constraints were imposed on the duration of the task nor on the strategy to pick up or turn the food. So, the subjects picked the most convenient way for them and, with tweezers, the subjects used slightly different approaches, as shown in Fig. 6.

The subjects were instructed to follow these rules: (1) do not help the movement with the second hand; (2) try not to hit the surrounding food while picking up or turning the food; (3) target the fall position to be the closest possible to the initial position.

#### Food samples

Different foods (that are usually grilled) were presented to the subjects: chicken-breast steak, hamburgers, eggplant slices and zucchini slices. Their characteristics are detailed in Table 3. In particular, when slicing the zucchini length-wise (*i.e*. longitudinally), we obtain two types of slices: *i)* slices from the internal part of the zucchini, that have peel only on the edges of the slice, and ii) slices from the external part of the zucchini, that have peel also on one face of the slice (like in Fig. 6a). External slices are less bendable than internal slices, because of the additional peel that external slices have. In addition, both faces of the internal slices are flat, while, in the external slices, the face with peel is convex. As these characteristics may influence flipping, we captured movements with both type of slices. Likewise, hamburgers also exist with different composition of protein and fat (which affect food adhesion to surfaces^30^), thus, two representative types were selected for this experiments: one fatter than the other.

**Table 3:** Characteristics of the food samples flipped by the subjects.

Food type	Shape	Notes	Mass	Dimensions
fat hamburger 21% fat, 18% protein	round	produced industrially; defrost before grilling	72.9 ± 5.2 g	Ø 120 mm × H 8 mm (±3 mm)
light hamburger (13% fat, 18% protein)	ellipsoid	produced industrially; defrost before grilling	103.1 ± 5.5 g	Ø 115 mm × Ø 83 mm × H 17 mm (±3 mm)
chicken breast steak	approximately rectangular, with irregularities	fresh, boned, trimmed and sliced in steaks	43.1 ± 7.1 g	80 mm (±10 mm) × 70 mm (±10 mm) × H 8 mm (±3 mm)
zucchini slices	ellipsoid/approximately rectangular (slices with peel have a convex face on one side)	fresh, longitudinal slices, with and without peel on one side - tip and middle slices, respectively	middle: 25.4 ± 9.4 g; tip: 25.8 ± 9.6 g	175 mm (±5 mm) × 30 mm × H 3 mm (±1 mm)
eggplant slices	round	fresh, sliced transversely	12.5 ± 4.9 g	Ø 95 mm (±5 mm) × H 4 mm (±2 mm)

The chemical composition of the hamburgers was provided by the producer. (H refers to thickness and Ø to diameter).

The authors registered the mass of the food that was flipped by the subjects. To do this accurately, the food cannot be weighed before cooking because food releases fluids while being cooked, losing mass. It also cannot be weighed immediately after cooking, *i.e*. before flipping, otherwise, the food may be damaged and, furthermore, it will not be stuck to the surface during the flipping movement. Therefore, all food samples were cooked, flipped by the subject and, only then, weighed.

The food was prepared for each experiment (*i.e*. sliced, trimmed, defrost, etc.) in the day of the experiment. The food was cooked under realistic conditions, while the experiment was being carried out: one of the researchers was with the subject performing the acquisitions and, simultaneously, a co-researcher was in the cooking station dealing with the food to cook.

#### Protocol description

When meat is cooked, it tends to stick to the metallic cooking surface, mostly due to its protein content^30^. To pick up meat, it is necessary to unstick it. Moreover, fat or water released by the meat during cooking spreads on the surface, as well as on the utensil (*e.g*. a spatula), making the meat piece slippery when it is moved (such as during the flip). Vegetable slices, instead, adhere poorly. On the other hand, they deform while cooking. These characteristics of adhesion, lubrication and shape deformation clearly affect the dynamics of flipping and how the subjects control that movement^1^.

So, to test the strategies that people use to control the flipping movements of grilled food, it is not sufficient to perform the experiments with plastic food models or with raw food. It is, instead, necessary to induce the aforementioned real food transformations and, thus, to cook the actual food, at least, on one side. Hence, to ensure repeatability among food and also due to time constraints, all food pieces were grilled only on one side. The hamburgers and the chicken were cooked for 1.5 min, and the slices of vegetables for 2 min, as the typical values to cook one side of these foods.

Specifically during the flipping movements, it is not necessary to be near the cooking appliance (*i.e*. the griddle), where it is very hot and humid and a sensor could be damaged or its measurements disturbed. Therefore, the movement (and the acquisitions) can be performed away from the griddle, if we ensure that the food characteristics created by cooking are preserved until the subject flips the food. Hence, to achieve this, a protocol was proposed in^1^ and implemented to collect this dataset: in summary, the food was not directly cooked over the griddle; instead, small metallic squared surfaces were placed over the griddle and the food cooked on them (see Fig. 1); this way, because the small surfaces (with the food on top) are movable, the food could be quickly moved by the researcher (in about 30 seconds) from the cooking station to the acquisitions station, without changing the characteristics of the cooked food. For efficiency and for greater similarity with real conditions, several pieces of food were cooked simultaneously and presented to the subject at once on two surfaces, with pieces side by side. Moreover, while a trial was being conducted in the acquisitions station, another batch of food was being prepared by a co-researcher in the cooking stations, keeping a flow of freshly cooked food in coordination with the trials workflow. It is also worth noting that the movable surfaces were placed on the table by fitting them perfectly in a support that was tailored for the surface dimensions. This ensured that the surfaces would remain still during the experiments.

**Fig. 7:**
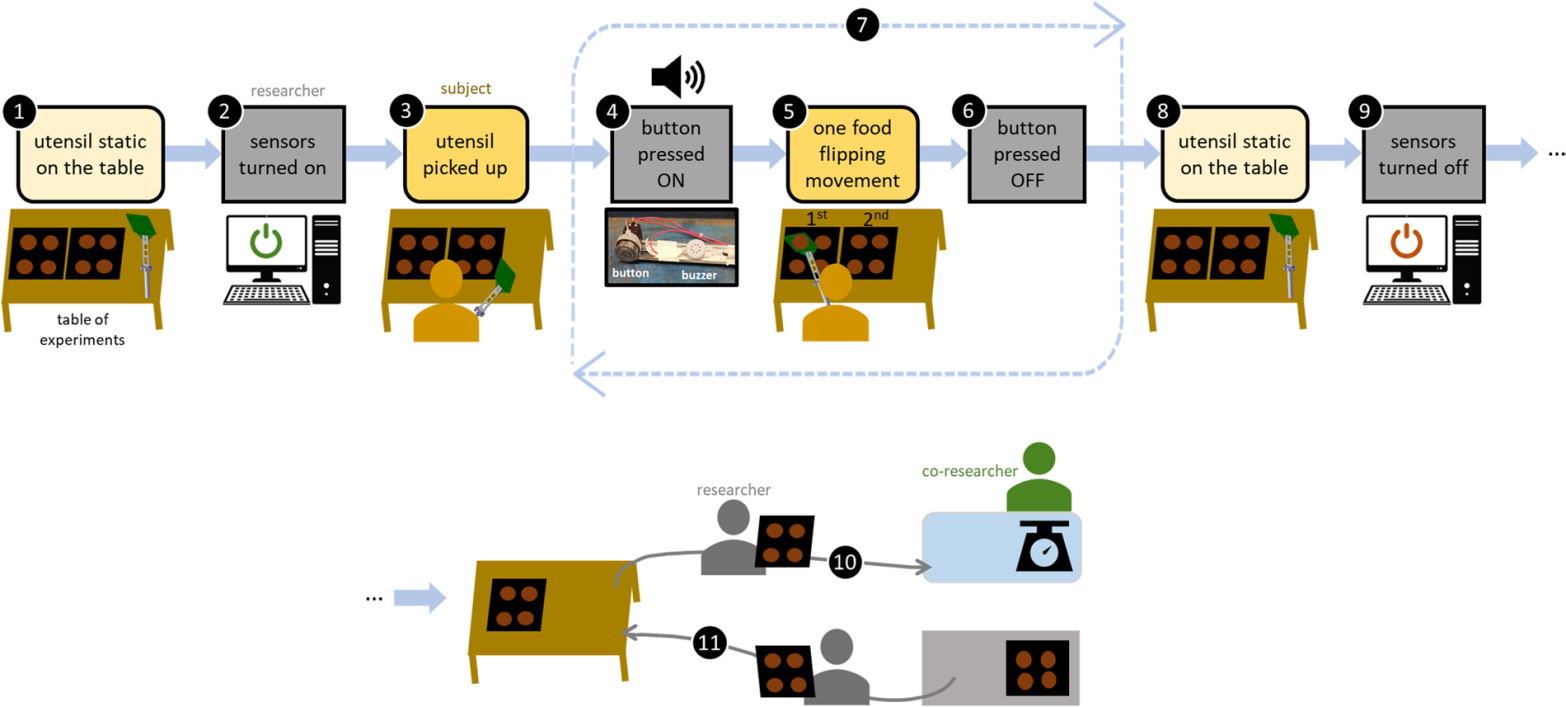
The steps of the experimental protocol, from 1 to 11.

The same researcher (first author) conducted the trials in the acquisitions station with all the subjects. The subjects remained always in the acquisitions station. Each trial was, then, conducted as follows (see also Fig. 7):

1. the utensil (spatula or tweezers) was on the table, static;
2. the researcher turned all sensors on;
3. the subject picked up the utensil and waited a warning;
4. the researcher pressed the button (ON), causing a buzzer to beep, that warned the subject to start a flipping movement;
5. the subject flipped a food piece;
6. at the end of the flipping movement, after the food fall, the researcher pressed the button (OFF);
7. the steps 4, 5 and 6 were repeated for each food piece, starting with the left tray and, then, the right tray;
8. after flipping all the samples on both surfaces, the subject would leave the utensil static on the table;
9. the trial finished, so, the researcher stopped all sensors and saved the data files;
10. the researcher took the two surfaces with flipped food to the co-researcher by the griddle who weighed the food;
11. the researcher picked up two new surfaces with freshly cooked food and brought them to the table for another trial.

Spatulas and tweezers are typically used in grilling tasks but, usually, not for all food. According to their common use, we acquired flipping movements where the subject used:

- a spatula to flip hamburgers, chicken breast, and the vegetable slices;
- tweezers to flip vegetable slices.

In the trials with vegetable slices, each slice was flipped more than once because they do not stick to the surface neither change their shape during the trial. This way, we avoided wasting more food. Before flipping again a given slice, the position of the slice was readjusted, if necessary.

Given the elongated shape of zucchini slices, we recorded trials with the slices placed along the y-axis represented in Fig. 3, and along the x-axis.

### Signal processing

#### MoCap data labelling and pre-processing

**Fig. 8:**
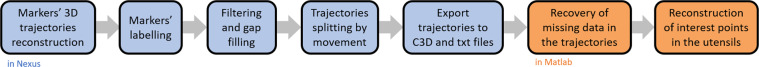
Flowchart of motion captured data elaboration. Blue boxes concern steps performed in Vicon Nexus software, and orange boxes concern steps performed in MATLAB. The interest points in the last block are the tips of the tweezers and the corners of the spatula blade.

The trajectories of the IR markers were labelled and pre-processed using the proprietary software of the motion capture system, *i.e*. Vicon Nexus (version 2.7.0, Oxford Metrics Ltd., UK). A skeleton template was created for each utensil and calibrated using the static trials. For each session with the utensil, the template was loaded and data were processed as follows (Fig. 8):

1. reconstruct 3D trajectories from 2D data;
2. label the first complete frame manually and perform the static skeleton calibration;
3. run the automatic labelling pipeline (the flow graph algorithm);
4. check the correctness of the trajectories in each frame, visually and by inspecting the inter-markers distance graphically;
5. manually switch or delete erroneous labelled markers;
6. fill small gaps (up to 10 frames) using the function “Woltring Quintic spline”;
7. fill longer gaps using the function “Rigid Body Fill” with markers of the same rigid segment of the utensil; this method was used to fill gaps no longer than 10% of the flipping duration - a percentage determined empirically to avoid sudden displacements or incorrect inter-markers distances.

Due to the rotatory trajectory of the flipping movement, often some markers were only partially visible to the cameras, especially during the last part of flipping (when the utensil is inverted) and more often with the tweezers’ markers. Therefore, the trajectories were further processed in MATLAB (version R2019b, Mathworks Inc., USA) to recover missing data, using more robust and advanced methods: we used the MoCap data recovery method from Tits *et al*.^31^, that is based on a probabilistic averaging of different individual MoCap data recovery models. This method was shown more robust than others and with low estimation errors, for different type of motion, gap length and motion sequence duration^31^. We selected it also for not requiring any prior knowledge or any pre-trained model and because the authors provide freely their implementation in MATLAB as an extension for the MATLAB MoCap toolbox developed by Burger *et al*.^32^.

In addition, in MATLAB, we reconstructed the 3D position of the key points in the utensils (points of major interest, defining the part of the utensil that comes in contact with food or a surface), *i.e*. the tweezers tips and the corners of the spatula blade, during the recorded movements. The trajectory of the tweezers tips may be relevant for the study of the flipping movements, *e.g*. to detect the instant when the tips touch or to track the inter-tips distance. Similarly, the three corners of the spatula blade can be useful to define the surface of the blade during the experiments and, *e.g*., to determine when it reaches the table or how it relates to the position of the food obtained from the videos. The position of the blade corners in the local coordinate system was obtained in the static trials. The local coordinate system is defined by the cluster of markers in the spatula segment connected to the blade. Then, in the recordings of the dynamic trials, the blade corners were reconstructed using the transformation matrix from the local coordinate system to the Vicon global system, obtained with the trajectories of the markers for each instant. The same was performed for the cluster of markers in each segment of the tweezers and the respective tip.

#### Video processing to track food

**Fig. 9:**
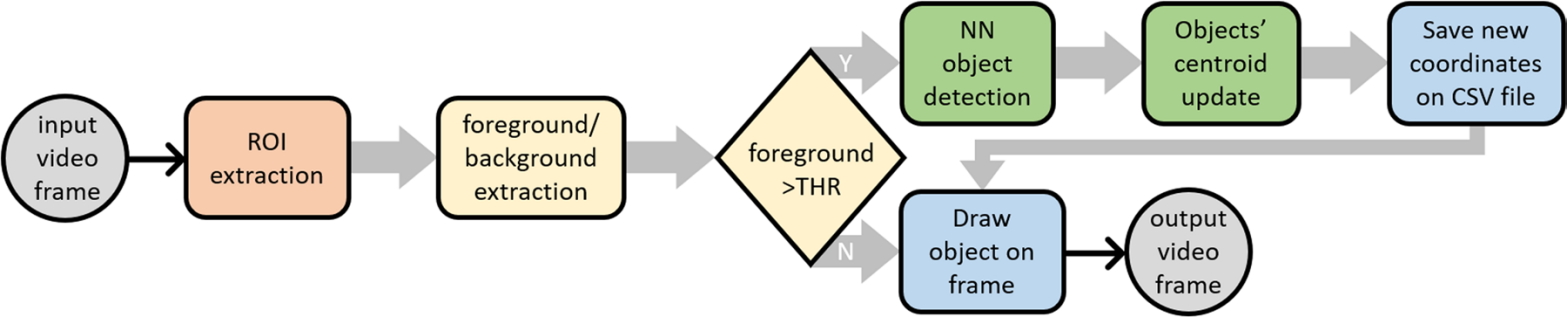
Video processing algorithm for food tracking. For each frame of the video a sub-matrix (ROI) is considered: the region of the experiments table. Afterwards, a foreground/background extractor counts the number of pixels belonging to the foreground. The neural network object detector is fed only with the frames having a foreground pixel count greater than a threshold (THR). The objects identified by the CNN are validated by a custom centroid tracker and their 2D position saved on a CSV file. Finally, all the information belonging to the identified objects is drawn overlying the frame in the output video.

A computer vision (CV) method was developed to track the 2D position of the food in the video records of the experiments. Five classes were tracked and labelled as: hamburger, chicken, eggplant, zucchini and marker. The latter corresponds to one of the infrared-reflective markers on the experiments table. It was tracked so that anyone interested can, later, match the 2D positions of food with the 3D positions of tools recorded with the motion capture system. Figure 9 shows an activity diagram of the developed CV method. The steps of the method (squared boxes in Fig. 9) are explained below:

1. **ROI extraction:** each frame of the video is “cropped” around the experiments table (the region of interest, ROI), as in Fig. 3, to prevent false detections of pieces of food outside of the table;
2. **Foreground/background extraction:** the frame is, then, passed to a foreground/background extraction algorithm^33^ that attributes a pixel to the background if the pixel was almost unaltered during the 300 preceding frames, or to the foreground otherwise;
3. **Foreground pixels quantification:** the number of foreground pixels in the frame is counted and compared to a threshold (THR); if the foreground pixels’ count surpasses the THR, the frame is processed by an object detector/tracker (step 4.); otherwise, step 4. is skipped and the frame keeps the labels and positions of the food pieces from the previous frame; a reasonable THR was pre-determined empirically by analysing several videos;
4. **Objects detection and tracking:** the selected frames are processed by an object detector (built on the well-known Mask R-CNN architecture^34^) and, then, processed by an object tracker based on centroids^35^;
5. **CSV data file update:** for each of the objects identified and located in the frame, one data row is added to a CSV file; the data in this row includes the 2D coordinates of the object and other data detailed in section “Food pose data files”;
6. **Output labeled video:** finally, the output frame (see an example in Fig. 3) is drawn by overlaying the identified objects (*i.e*., their ID, centroid and bounding box) over the original frame; this way, a labelled video is generated.

This method was applied to the complete dataset of videos in order to generate the trajectories of the food pieces for all the experiments. Further details are added bellow:

- **The rationale behind steps 2. and 3**.

These steps were performed because they allow one to save computational time in step 4. (which is long, given the high complexity of the detection/tracking algorithm and the huge amount of data): because it is more relevant to track food when a movement is happening, steps 2. and 3. ensure that only the frames that are captured during a movement are processed in step 4; in step 3., when the foreground pixels’ count surpasses THR, it means that the spatula was moving, so the respective pixels were attributed to the foreground increasing the count.

- **Training/test set preparation**
  The Convolutional Neural Network (CNN) was trained on a tiny set of 215 frames, to learn how to classify four food categories and one static marker. The frames in this set were randomly selected among the frames of the entire videos. The researchers labelled each visible object in the randomly selected frames and enclosed it in a bounding box, manually drawn. This labeling procedure generated an XML file in compliance with the PASCAL Visual Object Classes (VOC) format (see page 10 of Everingham *et al*.^36^). The set of frames was then subdivided into training (66%) and test (33%) sets^37^.
  The training/test set is also included in our code repository (in the folder foodFlippingDataset/foodTracking/food_dataset/).
- **CNN training procedure**
  Each frame belonging to the training set (and its corresponding XML file) was fed to the Mask R-CNN to train the head layers^38^. Training was executed over 10 epochs starting from the MS COCO pre-trained weights^39^.
- **Food position**
  The 2D position of the food was summed up to its centroid location. The centroid was calculated on the segmentation masks, instead of averaging the corners of the bounding boxes, because this improved its closeness to the real center of mass of the food.
- **Rules for the centroid tracking**
  Some further rules were added to the standard centroid tracker in order to minimize false movements and to reduce the errors coming from the object detection stage. For instance, by knowing the content of the videos, we imposed the rule to have only one food category in each video. Moreover, we allowed the objects to disappear from the scene for, at maximum, 800 subsequent frames, before dismissing them from the tracking queue. This detail affected mostly the food samples that are static on the table (*i.e*., that are not being flipped in that moment). These samples are frequently covered by the subject’s arm or the utensil and, thus, disappear from sight for several seconds. Hence, with the aforementioned rule, these samples can still be tracked after disappearing and, when they reappear, they keep the ID of the sample that had disappeared.
- **Bounding boxes in the CSV files**

Although the object detection algorithm directly outputs a bounding box (BB), this BB was discarded for being too approximate. Instead, the segmentation mask of the object (a second output of the algorithm) was used to re-calculate the object BB (defined as the smallest box containing the mask). Then, the masks were not saved in the CSV file, due to their large occupied space, but the calculated BBs were saved, together with the other data.

#### Processing of synchronization signals

- **Timestamps from the LSL LabRecorder**
  The stream of timestamps and letters presses were automatically extracted in MATLAB, from the XDF files produced by the LSL LabRecorder. The start/end of the acquisition programs was also automatically detected, being only preceded by a very simple manual step of “cleaning” the extracted streams: the authors observed the streams of pressed letters, identified letters that were not associated to the start/end of any acquisition program, and eliminated the respective timestamps. These few extra keyboard presses were evident and could be undoubtedly identified because their sequence corresponded to the name of data files, typed when the files were saved, at the end of a trial. These useless letter presses happened to be recorded because the LabRecorder was left running during several trials uninterruptedly, to facilitate the control of the other sensors. The quality of the manual “cleaning” step was verified by checking the time passed between the identified timestamps of the start and end of a program, and comparing it to the real duration of the corresponding signals (*i.e*. the Vicon signals, the eye tracking files, etc.).
- **Button signal**

This signal is simply a squared wave with levels ON (value 1, maximum) and OFF (value 0, minimum), with the subjects’ movements occurring inside ON levels. The button signal was not processed except for when a mistake occurred during the experiments and a minor correction was needed. These cases of a mistake, affecting only a few movements, included: (1) when a subject started the movement before hearing the beep (*i.e*., before the button was pressed ON by the researcher); (2) when the researcher forgot to press the button to OFF (*e.g*., at the end of a trial). In the first case, the real movement started before the ON level of the signal, so, offline, the start of this ON level was anticipated by some samples. In the second case, the end of the movement was not registered, so the OFF level was added to the signal. The instant in the signal, where to implement these corrections, was determined from analysing the bending/pinching force signals (with the support of the videos), because these signals allow a clear identification of the movements start/end (as observable in Figs. 4 and 5). Note that these corrections were performed offline, in MATLAB, thus, the C3D files still store the original button signal while MAT files store the corrected button signal.

MoCap data, eye tracking data and videos were synchronized using the difference between the timestamps of their respective program start. Then, the button signal recorded together with the MoCap data was aligned with the button signal recorded with the forces/torques, by making their ON/OFF periods coincident. This way, forces/torques and MoCap signals were synchronized, and the synchronization of all the data was achieved.

#### Data splitting and size reduction

As explained before, each trial included the execution of several flipping movements without stopping the signals’ acquisition, to facilitate conducting the experiments. However, data analysis and data visualization are enhanced if the signals are split per movement. Hence, offline, MoCap data (raw and pre-processed), eye tracking data and force/torque signals were split in several files, one for each movement.

To do so, after having all the signals synchronized and, thus, aligned with the button signal, the ON/OFF switches of the button were used to split the data. More specifically, to keep some pre and post-movement information, all signals were cut half second before the button was pressed ON (or less, if the preceding movement ended less than 0.5 s before) and half second after the button was pressed OFF (or less, if the next movement started less than 0.5 s after). Note that when two consecutive movements were performed with less than one second in between, the signal extremities appear in both of the files.

MoCap data were split in Nexus using the “Export C3D” and “Export ASCII” operations. A MATLAB script was created to automatically interact with Nexus, select the start/end frames, and run the export operations, for each recorded movement.

The eye tracking data of interest were, first, exported from the original recordings, using the “Pupil Player” tool provided by Pupil Labs. These data include the pupil and gaze positions, surfaces location, and the world video having the eye, the pupil detection and the gaze circle overlaid. To export such data, the following plugins were activated in Pupil Player: “Eye overlay”, “Raw Data Exporter”, “Surface Tracker” and “World Video Exporter”. Then, the resulting CSV files were automatically split in MATLAB, and the world videos were split in Python using the OpenCV VideoWriter class.

The original videos that record the experiments table and the output videos of the food tracking algorithm were not split, but only trimmed in their extremities (with the aforementioned Python class) so that the start of the video corresponds to the start of the first movement in the rest of the acquired signals. Then, with the open-source VLC media player (version 3.0.11, by VideoLAN), the videos were converted to the H-264 codec (*i.e*. the video compression standard MPEG-4 AVC part 10). These operations were performed to reduce the videos size (initially, hundreds of GB in total). All videos are shared in MP4 format.

#### Movements labelling as successful/unsuccessful

**Fig. 10:**
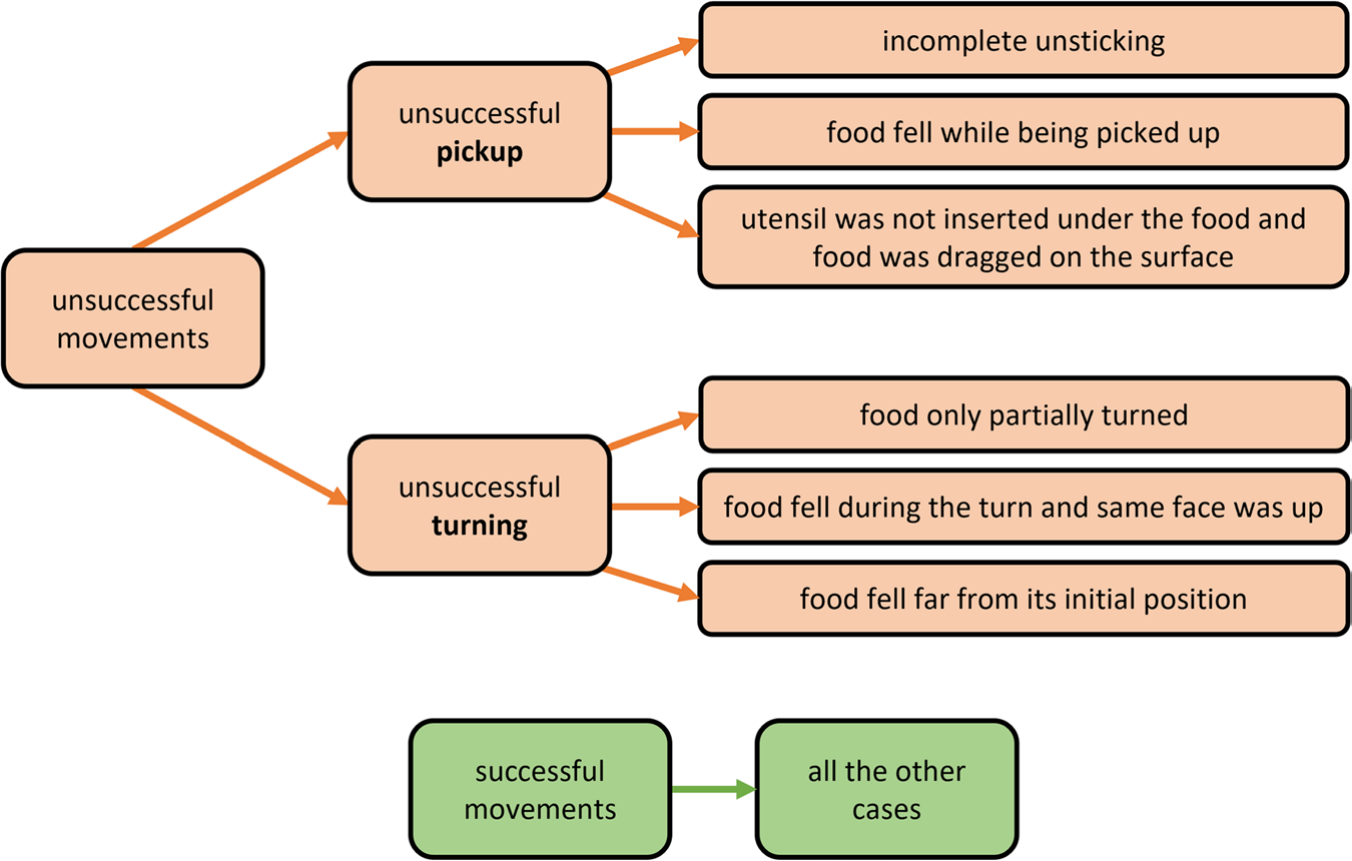
The suggested definition for successful/unsuccessful movements.

Even if the subjects had time to practise and made an effort to perform the movements correctly, they did not always succeed in picking up or in turning the food – we recall that a flipping movement consists on picking up the food (that may involve unsticking it) and turning it upside down. We recorded both successful and unsuccessful movements as they may be both useful for different studies. We suggest a definition for successful/unsuccessful movements, that researchers are free to adopt or not. For an unsuccessful movement, either an unsuccessful unsticking/pickup or an unsuccessful turn occurred, as defined below (and in Fig. 10):

- *pickup/unsticking*: if the subject raised the food and initiated the turning phase when the food was still stuck to the surface, then, unsticking was not done completely and it is considered an unsuccessful unsticking; in case of food that does not stick to the surface (vegetables), if the subject picked it up and it immediately falls or if the subject was not able to insert the utensil below the food and dragged the food out of its position, thus, pickup is considered unsuccessful; otherwise, pickup/unsticking is considered successful (even when the subject applies several strikes to unstick food, before raising it to turn).
- *turning*: if the piece of food was only partially turned (as it can happen with *e.g*. zucchini slices for being soft), this is considered an incomplete and, thus, unsuccessful turn; if the food falls during the turning phase and its position was not inverted, this is considered an unsuccessful turn; if the food falls considerably far from its initial position (*i.e*., with the food centroid deviated by 20 mm or more, with respect to its initial position), this is also an unsuccessful turn; otherwise, the turn is considered successful.

For some unsuccessful movements, the subject was allowed to repeat the movement. With meat, the second attempt was stored as it may be useful for some studies, but it was labelled unsuccessful independently of the outcome, given that, in the second attempt to flip meat, this is already unstuck so the meat is not in the same conditions as in the other movements. This does not happen with vegetables, which usually do not stick. So, with vegetables, if the movement repetition succeeded, it was accounted as a new movement and labelled successful.

According to these definitions, we reviewed all the movements in the videos and, for each subject, we created a file with the list of movements performed by that subject and their corresponding “success” or “fail” label. For better comprehension, the “fail” labels are accompanied by a justification (*e.g*. “failed unsticking”, “incomplete turn”, “second attempt, food not stuck”). Only about 15% of all movements in the dataset are labelled with “fail”, being the tip slices of zucchini the most common food among failed movements.

## Data Records

The dataset here described is stored in figshare^40^. All data were de-identified by converting the subjects name to an index (*i.e*. S1, S2, etc.), used in the name of folders/files as well as within the shared code.

### Volume of data collected

A total of 2866 flipping movements (1895 with spatula and 971 with tweezers) were recorded across all the subjects and conditions (*i.e*. different food) – find more details in Table 4. These data were already used in the analysis of^1^, except for the trials where the zucchini slices were placed along the y-axis (Fig. 3), because the curation of these data and their inclusion in the dataset was an afterthought.

**Table 4:** Volume of data collected.

Number of movements recorded with	Spatula						Tweezers		
	fat hamburgers	light hamburgers	chicken breast	zucchini middle slices	zucchini tip slices	eggplant slices	zucchini middle slices	zucchini tip slices	eggplant slices
chefs	97	98	98	187	190	102	112	118	96
not-chefs	121	129	123	272	316	162	198	286	161

### Data files formats and organization

**Fig. 11:**
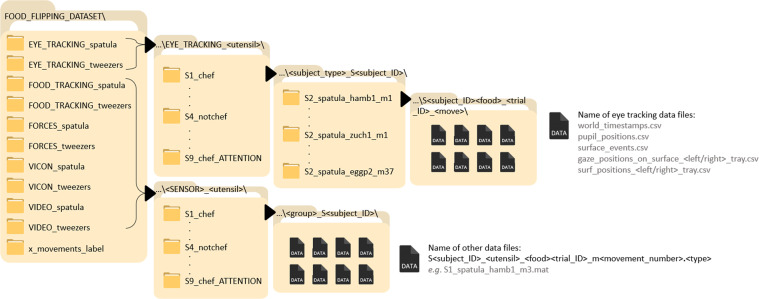
Dataset organization: folders structure and filenames format.

All data are shared in standard file types (or types in common use, such as MAT) and most of them are open file formats (*e.g*. TXT, CSV, MP4). The name of the data files indicates the subject ID, the utensil used, the food flipped, the trial ID and the movement number - see Fig. 11. In the filename of the videos, there is no movement number as they are not split by movement.

#### Folders structure

The dataset was organized in a way that simplifies its download for different goals: the main folders of the dataset are separated (1) by data type (*i.e*. kinematics, force/torque, videos, food pose and eye gaze), and (2) by utensil (spatula, tweezers). Inside each of these folders, there is one folder per subject that contains the data files for all the movements performed by that person. The name of these folders indicates the subject group (*chef* or *notchef*) and the subject ID (*i.e*., S1, S2, etc.) - see Fig. 11. This structure allows a researcher to download data only for the study of *e.g*. kinematics or dynamics, or only for one type of utensil, selecting the respective folder, without having to download all the data.

The eye tracking folders have a small difference because, for each movement, there are several files that regard different data. Hence, inside the subject folder, there is one folder for each movement with all the corresponding files. These files are named with the data they store (*e.g*., “pupil_positions.csv”, “world.mp4”).

Success/Fail movement labels are saved in CSV files named “success_labels_S<subject_number>.csv”, in the folder “x_movements_label”.

#### Motion captured data files

In the dataset, we share the MoCap data in three file formats:

- C3D - these files contain the 3D trajectories of the IR markers, processed in Nexus (*i.e*. labelled and corrected regarding label switches and gaps), as well as the analog signal of the button voltage (as originally recorded, without the corrections explained in “Processing of synchronization signals”) - see Fig. 4a; this is a public binary file format supported by motion capture systems and biomechanics software^41^, so, these files are useful for visualization or to perform additional pre-processing in software like Nexus.
- ASCII - these files also store the 3D trajectories but in a simple format that can be loaded into most software for post-processing.
- MAT - these files contain the output from the data elaboration in MATLAB, *i.e*. the trajectories of all markers without gaps (struct variable *recovered_trajectories* with a field for each marker trajectory) and, according to the utensil used in the trial, the trajectories of the tweezers’ tips (struct *tips_position* with fields *arm_loadcell* and *arm_no_loadcell*) or the corners of the spatula blade (struct *blade_corners* with four fields, one for each corner); in addition, the corrected button signal is also saved (array *button*).

Inside the folder of one subject, there are files regarding dynamic trials and files regarding static trials. For each movement of a dynamic trial, there is one C3D file, one ASCII file and one MAT file. Their filename follows the format in Fig. 11. For the static trials (gravity vector and food surfaces), there is only the ASCII file, with the filename in the format “S<subject_ID>static_<object>.txt”, like *e.g*. “S1_static_gravity.txt”. The abbreviation for the object can be the “spatula”, “tweezer”, “trays” (for the right/left surfaces) or “gravity” (for the gravity-vector). Additionally, we provide the VSK file with the utensils model template to be used in Nexus with the C3D files, so that one can better distinguish the markers by their color.

#### Force/torque data files

Force, torque, temperature and button voltage signals acquired with Simulink are shared in a single MAT file. As with MoCap data, there is a file for each movement performed by the subjects. Note that a termination “_f” was added to the name of these files (*e.g*. “S1_spatula_hamb1_m1_f.mat”), to avoid confusing them with the MAT files with the same name that are in the folder of MoCap data. The file contains 9 variables as follows:

1. *pinching_force* - struct with fields.*raw* (an array with the analog readings of the load cell in the tweezers) and.*converted* (an array with the pinching force, in Newton, converted with the respective calibration curve);
2. *bending_moment_A* - struct with fields.*raw* (array with the analog readings of the Wheatstone bridge A in the spatula) and.*converted* (array with the bending moment in A, in Newton.metro, converted with the respective calibration curve);
3. *bending_moment_B* - like the variable “bending_moment_A”, but for the Wheatstone bridge B;
4. *bending_force* - array with the bending force in the spatula, calculated with Eq. 2;
5. *torsion_moment* - struct with fields.*raw* (array with the analog readings of the torsion-sensing Wheatstone bridge in the spatula) and .*converted* (array with the torsion torque, in Newton.metro, converted with the respective calibration curve);
6. *temperature* - struct with fields.*top* and .*bottom* – arrays with the temperature readings from the thermocouples, respectively, at the top and bottom of the spatula beam;
7. *button* - array with the button signal already binarized (*i.e*. a square wave with 0 and 1 levels, meaning OFF and ON, respectively), and already corrected as explained in “Processing of synchronization signals”.

In trials with the spatula, the variable “pinching_force” is a static signal (only with noise); conversely, in trials with the tweezers, the signals 2. to 5. are static.

#### Food pose data files

The data in the CSV file were written in the following format: iteration number, frame number, object ID, object category, object centroid x-coordinate, object centroid y-coordinate, object area, x-coordinate of the left-top corner of the bounding box, y-coordinate of the left-top corner of the bounding box, x-coordinate of the right-bottom corner of the bounding box, y-coordinate of the right-bottom corner of the bounding box, identification precision. Some notes are left below:

- the object area corresponds to the area of the segmentation mask that was predicted by the algorithm for that object;
- all the data are reported in pixels. Distances and measurement conversions (pixel to meters) can be easily calculated starting from the average area of the pink square on the table, visible in Fig. 3, which is 76.5 × 76.5 mm;
- the “iteration number” identifies the *i*th time that the food detector/tracker was applied - we remind the readers that the food detector/tracker is not applied to all the frames, as explained in step 2 of the section “Video processing to track food” (for this reason, “iteration number” is not always equal to the frame number and the total number of iterations is not the total number of frames);
- number used for each object category: chicken - 1; hamburger - 2; zucchini - 3; eggplant - 4; IR marker - 5.

#### Eye tracking data files

In addition to the CSV data files and the videos, the reader will find, inside the dataset main folder, one ASCII file (“pupil_gaze_positions_info.txt”) that defines the variables written the CSV files.

#### Instructions to download the dataset

Interested readers can access the DOI link to *figshare*^40^ in order to retrieve the data here described. In this repository webpage, under the title of the dataset, the reader will find various clickable sub-titles that correspond to folders of the dataset.

The title/name of a folder indicates the data type (*e.g*., eye tracking data) that the folder contains, as well as the utensil (spatula/tweezers) used by the subject when such data was acquired (*e.g*., one folder is named “Eye tracking data (spatula)”). To download a specific folder, the reader must click on the folder name and, in the next page, click on the symbol to download the zip file (images/download_file_symbol.png).

To download the whole dataset, the reader must repeat these steps for all the folders that are in the main page of the dataset.

## Technical Validation

### Data quality

**Fig. 12:**
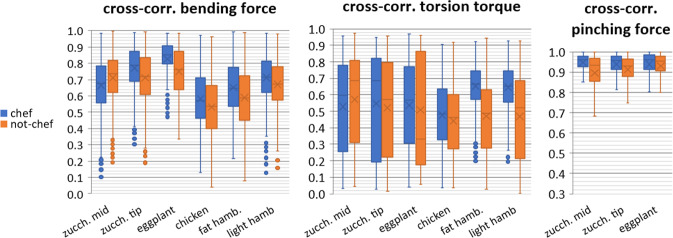
Repeatability of the forces and torque signals across flipping movements performed by 4 chefs and 5 home cooks (“not-chefs”): cross-correlation between repetitions of flipping a same food (box plots with average values, standard deviation and outliers). See the exact number of movements for each condition in Table 4. Note: this figure includes the graphs of Fig. 10 from our related work^1^.

The repeatability of force/torque signals profile was assessed. Bending and pinching force profiles are visibly similar across repetitions of flipping movements (as one can observe in Fig. 5). There is a comparable trend for *chefs* and *not-chefs*. Furthermore, the high cross-correlation (CC) between force profiles along repetitions with the same food confirms the similarity for most food types, as shown in Fig. 12: mean CC > 0.6, except for chicken. Some subjects tried to unstick chicken with several hits (see, *e.g*., the flip with ** in Fig. 5) instead of a single stroke (flip with *, Fig. 5). This is a possible reason for the reduced repeatability with chicken. Finally, the CC of torsion signals is, in average, lower for most food (except hamburgers flipped by *chefs*) and highly variable (standard deviations up to 0.3).

**Fig. 13:**
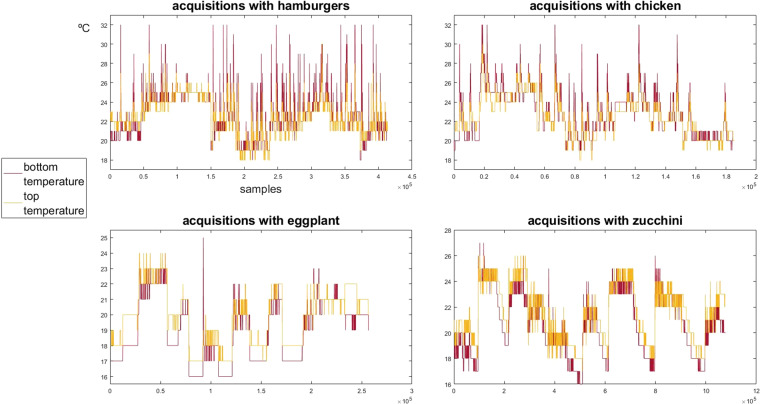
Temperature measured near the strain gauges of the spatula (in the bottom surface and in the top surface of the beam) during all the acquisitions time. The plots show the temperature signals concatenated across experimental sessions.

To what concerns the temperature observed near the spatula sensors, during the experiments, one can note, from Fig. 13, that the temperature was in a safe range not to disturb the measurements:^1^ always under 32 °C.

We also recall that a deeper assessment specifically of the custom utensils with force/torque sensors was previously done by Pereira *et al*. in^1^, that included the noise, cross-talking and linearity of the sensors response.

Regarding the MoCap data, we determined the distance between markers in a same rigid segment of the utensils, along all the recorded movements (after all the post-processing), as a reference for the position tracking error. In average (*i.e*. for the different pairs of markers), the inter-markers distance varies 1.3 mm (standard deviation), in the spatula, and 2.1 mm, in the tweezers, with respect to its real value. This indicates that the trajectories are affected by an acceptable error level, similarly to other datasets with MoCap data (*e.g*.^42^). Furthermore, the calibration of the system, in the days of experiments, always showed a good quality for its typical performance^43^: a mean absolute error of 0.06 mm was obtained across different calibrations with a standard deviation (inter-calibrations) of 0.003 mm. These values were calculated from all the calibration errors of different days/experimental sessions.

To what concerns the eye tracking, we recall that, to ensure a good data quality, data were collected always after a calibration with a high accuracy, i.e. with, at maximum, 2 degrees of residual between reference points and matching gaze positions recorded during calibration. Another point is that, before the start of every trial, the subject was asked to indicate where he/she was looking to confirm the correctness of the gaze estimation. Even though, we encountered a considerable challenge with the eye tracking setup because, with several participants, the pupil was often obscured. This happened for several reasons: subjects had long eye lashes, would blink frequently or move their head very suddenly and frequently, or they would keep their head elevated and the eyes looking down to the table (resulting in partially closed eyelids). This last situation was found very common among subjects: although the eye and world cameras were adjusted before the trials, according to the subjects characteristics (*e.g*. lowering the eye camera in case of long eye lashes), it was not enough to have the pupil always visible when the subjects were looking down with their head elevated. We also noted that illumination in the room was greatly influencing the ability of the algorithm to detect and keep track of the pupil, even in situations where the pupil did not seem hidden. The insufficient lighting was possibly causing a low contrast between iris and pupil, making its detection more difficult. For these reasons, the pupil was often tracked erroneously. With a confidence higher than 0.3, the pupil was tracked for the following percentage of flipping movements (in their complete duration): 91% of the movements of S1; 61% of S2; 58% of S3; 72% of S4; 0% of S5; 87% of S6; 21% of S7; 91% of S8; 14% of S9. Despite the incompleteness of these data, we decided to share it as it may be useful for preliminary studies.

### Validation of the synchronization method used with subject S9

The 3D trajectories and force/torque signals are synchronized by calculating the delay between them and, then, using this delay to align the signals in time. The data of subject S9 had to be synchronized differently from the rest of the data, so, here we present the validation of this alternative synchronization method.

We recall that the data of S9 was synchronized by detecting the abrupt variation of both the 3D trajectories (MoCap data) and force/torque signals at the beginning of each trial, caused by the subject when picking up the utensil from its static position on the table. More specifically, we searched an abrupt variation of the signals’ mean and slope, applying the MATLAB function *findchangepts* (with ‘linear’ statistics)^44^ to the initial part of the signal (*i.e*., before all the flipping movements). Then, the signals were visually inspected to verify the output of the function. This procedure was meant to replace the button signal, since it was missing that day.

Hence, to validate this approach, we applied it to the data of trials with subjects S1-S8 and, then, we compared the outcome of this method to that of the nominal approach (*i.e*., that with the button signal). Results, in terms of number of samples, revealed that the median (and IQR) difference between the two approaches across subjects and trials was 3 (5) samples, roughly reflecting a time-shift of 0.01–0.06 s (quartile 1 - quartile 3).

The distribution of the error for the several trials can be observed in the Fig. 5 of the document in “Supplementary Information”.

### Performance of the food tracking algorithm

The performance of the algorithm, in its current version, was assessed on a test set with 160 images (40 images per food class – hamburger, chicken, eggplant, zucchini). The test set was obtained by randomly selecting frames from the videos of the entire dataset. This test set is representative of the complete dataset as the various random frames captured different instants of the flipping movements (and, thus, different perspectives of the food) as well as static parts of the videos.

**Fig. 14:**
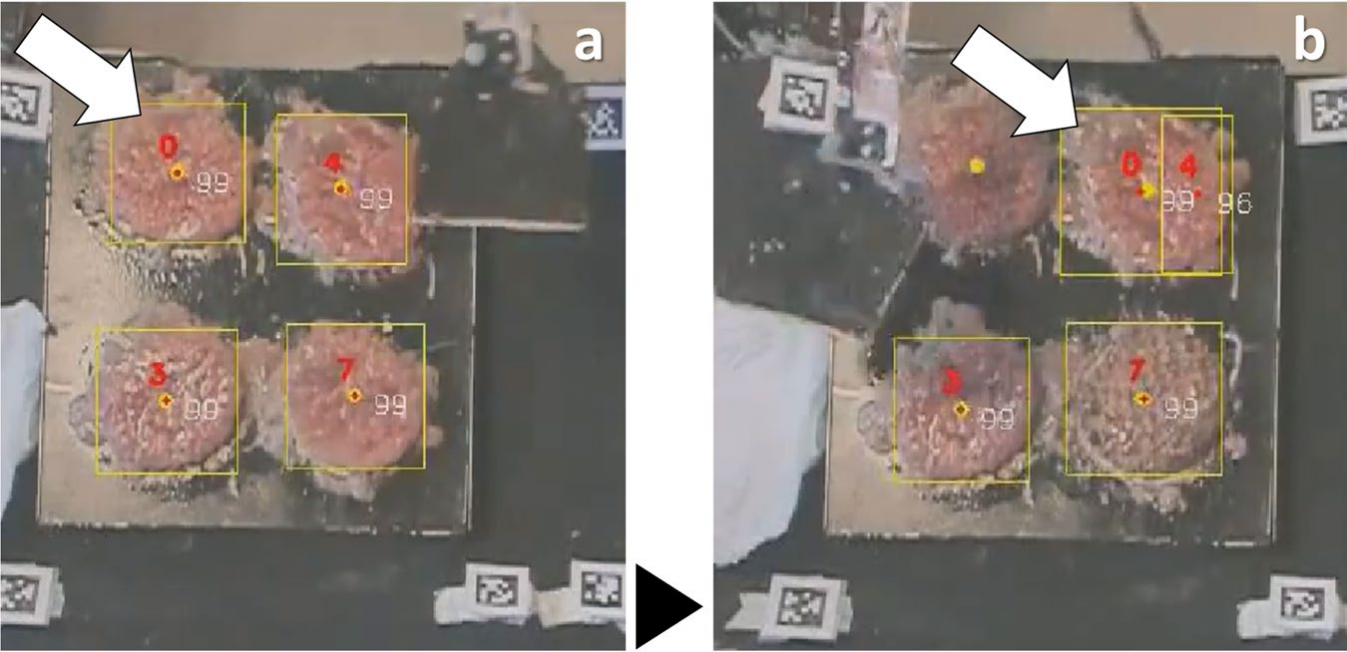
Example of an ID switch: (**a**) the detected object 0 was first matched with the ground truth (GT) hamburger at left (*i.e*. the detection 0 was considered a true positive and it was associated to that specific GT) and, (**b**) later in the video, the detection 0 was matched with the GT hamburger at right; so, the hamburgers switched their ID.

The assessment was done by selecting the frames, running the algorithm over the full dataset (because, e.g., the fore/background extraction depends on consecutive frames), collecting the outputs for the selected frames, and calculating six standard performance metrics that are currently used in machine learning, to assess object detectors^45–47^ and trackers:^48–52^ accuracy (Eq. 3, as in^36^), precision (Eq. 4^46^), recall (Eq. 5^46^), F1 score or F-measure (Eq. 6^47^), centroid position tracking error (PE, as in Eq. 7, *i.e*., the position tracking error between the ground truth centroid and the estimated centroid of the detected food)^48–50^, and ID switches (*i.e*., the number of switches of the detected objects’ ID, considering only TP detections^52^, as the example in Fig. 14). While precision measures the ability of a model to identify *only* relevant objects, recall measures the ability of a model to find *all* relevant objects^46^. F1 score is the first harmonic mean between precision and recall^47^. These metrics evaluate the capabilities of an algorithm both to classify the objects and to track them, because the judgement as true/false positive/negative depends on the overlap between the true/output bounding boxes^36^ and, thus, on the position of the bounding boxes. Following the common choice of previous researchers^36,45^, we considered a positive detection when the overlap area exceeded 50% of the union of the true/output bounding boxes area (i.e., a threshold of 0.5 for the intersection over union ratio, or IoU^46^).

3 $${\rm{accuracy}}=\frac{{\rm{TP}}}{{\rm{TP}}+{\rm{FP}}+{\rm{FN}}},\;{\rm{with}}\;{\rm{TP}}={\rm{true}}\;{\rm{positives,}}\;{\rm{FP}}={\rm{false}}\;{\rm{positives,}}\;{\rm{FN}}={\rm{false}}\;{\rm{negatives}}$$

4 $${\rm{precision}}=\frac{{\rm{TP}}}{{\rm{TP}}+{\rm{FP}}}=\frac{{\rm{TP}}}{{\rm{number}}\;{\rm{of}}\;{\rm{all}}\;{\rm{detections}}}$$

5 $${\rm{recall}}=\frac{{\rm{TP}}}{{\rm{TP}}+{\rm{FN}}}=\frac{{\rm{TP}}}{{\rm{number}}\;{\rm{of}}\;{\rm{all}}\;{\rm{ground}}\;{\rm{truths}}}$$

6 $${\rm{F1}}=2\cdot \frac{{\rm{precision}}\times {\rm{recall}}}{{\rm{precision}}+{\rm{recall}}}$$

7 $$\begin{array}{l}{\rm{PE}}=\frac{{\sum }_{N}\left\Vert po{s}_{det}-po{s}_{GT}\right\Vert }{N},{\rm{N}}={\rm{GT}}/{\rm{detection}}\;{\rm{matches,}}\\ {pos}_{det}={\rm{detected}}\;{\rm{centroid}}\;{\rm{position,}}\;\;{pos}_{GT}={\rm{GT\; centroid\; position}}\end{array}$$

The results of the test are presented in Table 5. The IR marker is always detected by the algorithm in all frames (always one marker per frame), so the first four metrics for this extra class are equal to 1 and the ID switches and PE are 0.

**Table 5:** Performance of the food tracking algorithm, evaluated in a test set of 160 representative images.

		metric					
		accuracy	precision	recall	F1 score	ID switches	PE (mm)
class	chicken	0.879	0.935	0.935	0.935	0	4.85 ± 3.35
	hamburger	0.918	0.940	0.975	0.957	1	4.63 ± 2.91
	zucchini	0.891	0.931	0.954	0.942	3	8.30 ± 4.77
	eggplant	0.994	0.997	0.997	0.997	0	3.79 ± 2.64
	AVERAGE	0.921	0.951	0.965	0.958	1	5.14 ± 3.42

The characteristics of the utensils played a fundamental role in the accuracy of the tracking algorithm: whereas the tweezers allowed a constant (even partial) visibility of the food pieces, the spatula, most of the times, covered the food in the final part of the flipping movement, occluding the vision system and, thus, increasing the error in the estimation of the food position. In these cases, further data processing may be necessary. For example, although less accurate, the trajectory may be interpolated from the point where food is not seen (usually during the falling phase) up to the next visible position (when the food is already on the surface).

Performances are not equal among categories: whereas chicken and eggplant food pieces are well identified and tracked, hamburgers and zucchini suffer from different detection problems.

Concerning the hamburgers, the object detection algorithm is not trained to recognize the spatula, the tweezers or the subject’s hands/arms since they do not belong to any category. Thus, sometimes the spatula is detected as an hamburger for having multiple shared features with that category, like color, shape and texture, when fat is present over the spatula surface (released by the hamburgers). This problem could be addressed by assigning the spatula to an additional class. However, it would be necessary to relax some constraints of the algorithm to allow the identified objects to be overlapped (like when the spatula covers the food). For the moment, the authors considered this issue to be of minor importance and the error small enough to allow sharing this version with the scientific community.

The zucchini, instead, suffer from multiple issues: they acquire diverse shapes with cooking and may have different colors due to the presence or absence of peel (middle vs. extremity zucchini slices). The darker green slices (those with peel) are hard to distinguish from the black background of the surface and table. Moreover, the distance metrics we imposed in the centroid tracker rules, sometimes, are violated by deformation of the zucchini slices (for being soft objects), which results in erroneous tracking.

### Dataset completeness

The integrity of all the data was checked before sharing this dataset. Here, the missing data and known causes are listed.

Concerning the motion capture, the IR markers were placed on the utensils and the cameras were arranged in such a way that optimized the visibility for tracking while not disturbing the subjects. Nonetheless, some occlusions could not be avoided due to the task itself, *i.e*. because the utensil rotates approximately 180° to invert the food piece, and the utensil intermittently approaches the table, which inevitably occludes the view from below. This difficulty was especially noticeable with the tweezers, in which the space to place the markers was more constrained. These gaps are visible in the C3D and ASCII files. However, by using the method of^31^, it was possible to recover the full trajectory of all markers in all the recorded movements and, consequently, the complete trajectory of keypoints in the utensils (*i.e*. tweezers’ tips and spatula blade corners). So, in the MAT files, there are no trajectory gaps within the movements duration.

Regarding missing files, we verified that three files were corrupted due to an error in the process of saving them. Thus, they were excluded from the dataset: (1) the video of subject S9 in the third trial with hamburgers (“hamb3”) that included 8 flips; (2) the MAT file with forces/torques recorded with S7 in the first trial with zucchini slices (“zuch1”), that included 31 flips of middle slices placed along the x-axis; and (3) the MoCap data of subject S1 in the first trial with hamburgers (“hamb1”) that included 8 flips. Still, the data from the other sensors for these trials are shared, so, these movements were accounted in the numbers in Table 4. For visualization purposes, the world video from the eye tracker can compensate for the missing video of S9. Additionally, Vicon system was not initiated, by mistake, in the second trial of subject S2 with eggplant slices (“eggp2”) that included 37 flips. This was detected in the experiments, so, a third trial with eggplant (“eggp3”) was performed with subject S2 (33 movements). Although the MoCap data are not available for the trial “eggp2”, the signals of the remaining sensors are available.

### Limitations

One should note that, other than food type and subject experience, more factors can influence food stickiness and, consequently, flipping movements. These other factors include, for example: the addition of spices, salt or oil; the use of coated surfaces or different metals; the cooking time; the initial state of the food (fresh vs. frozen); or the cleanliness of the grill. These factors were not explored in these experiments and were kept constant along the trials: the same cooking time was set for all the trials; fresh food was used, as noted in Table 3 – in case of originally frozen food, like the hamburgers, this was defrosted before the trials; food was not seasoned; all the squared surfaces where the food was grilled and flipped were made of the same material and were washed and dried before placing the food.

Researchers should also note that a dynamic analysis of flipping movements using this dataset will miss the effect of compression forces. More effort will be necessary in future experiments to succeed in collecting these signals.

Another point is the fact that human arm/hand motion was not included in this dataset. The main interest was in capturing the dynamics (kinematics and kinetics) of the utensil (that is the end-effector in food flipping), independently of the joints/degrees-of-freedom (DoFs) of the arms and hands of the human. This dataset is suitable for studies of the flipping movement at the end-effector level, for example, with the final aim of replicating the utensil movements in an automatic system (not necessarily having the same number of DoFs of the human arm), or with the aim of teaching human apprentices to flip food – applications in which it makes sense to characterize the movement of the end-effector/utensil rather than the movement of the human arm/hand. This dataset is not indicated for researchers wishing to study the contribution of the particular set of joints (DoFs) of the human arm/hand to the final food flipping movements.

Finally, the reader must be aware that the videos were not recorded with a real-time system and, therefore, the acquisition frequency varies to some extent along the recording. As so, although the beginning of the videos is precisely aligned with the beginning of the other signals, the timestamp of each frame in the videos must be obtained in order to match them with each sample of the other signals. This can be done with the time displayed by the millisecond-clock on the table. This may be in the interest of a researcher that intends to merge the information from the food tracking with MoCap data, for example.

## Usage Notes

To generate the food pose in our dataset, the food tracking algorithm was run on a Dell Precision 5520 computer having an NVIDIA Quadro M1200 GPU. Given the small size of the GPU memory used (4GB), the algorithm took several hours to process each video. If the algorithm is to be reused by any researcher with videos of similar size as those in our dataset, or to re-obtain, for any reason, the 2D positions from the videos in this dataset, the authors advise using a more powerful GPU memory and, thus, setting a greater batch size in the detection algorithm.

Concerning the button voltage signal, we recall the reader that such signal works only as a guide to know when the movement occurred, but the exact start and end of a movement do not coincide with the start/end instants of when the button is ON. These should be determined, within the time in which the button is ON, using the dynamic signals, *e.g*. using the bending force signals because their shape visibly reflects the movement phases.

The number of subjects in the dataset is not very high by a statistical point of view, so, we advise researchers to assess the statistical power and effect size of their results when analysing these data^53^. Up to now, it was not possible for us to collect data from more subjects given the current pandemic related to COVID-19. In addition to the thorough description of the protocol and setup presented in the methods section, the instructions for reproducing the instrumented utensils is fully described in^1^, so that any interested researcher can replicate the experiments and collect more data in the future. Authors are available to support researchers in reproducing and replicating the presented tools and tests.

Despite the current number of participants, to our knowledge, this dataset is currently the only one publicly available (1) that, within food manipulation, focuses on the specific and complex action of flipping food (which is in the interest of various industrial applications), (2) that has been performed not only by regular people but also by a specialized group of expert cooks, and (3) that has been recorded under a realistic scenario with real and varied food. All these conditions made the tests very complex but, for the readers, very precious to have and use as the starting point for their studies.

## Supplementary information

Supplementary Information

## Data Availability

The authors make available the custom code: • written in MATLAB, to obtain the trajectories of points of interest in the utensils, from the motion capture data; • written in MATLAB, to display the movements in a 3D plot. • written in MATLAB, to interact with Nexus and automatically split C3D and ASCII files, because this may be useful for any study that requires processing a huge amount of MoCap data; • written in Python, to track food in video records; • written in Python, to calculate the metrics to assess the performance of the tracking algorithm. • written in C++, to synchronize the clocks of the two computers, that was run before every experimental session. All the code is freely available on the following repository: https://github.com/deborapereira/foodFlippingDataset. More detailed information on how to use it is embedded in the code files. The code to process the videos uses Python 3.6.4, OpenCV 4.1.2, Keras 2.2.4 and Tensorflow 1.15.0. The code for the mask R-CNN can be found at https://github.com/matterport/Mask_RCNN/releases/tag/v2.1. The version 2.1 was used with this dataset. The code to calculate the performance metrics was adapted from https://github.com/rafaelpadilla/Object-Detection-Metrics, a tool developed by the authors of^46^.
