# Supplementary material for: Flipping food during grilling tasks, a dataset of utensils kinematics and dynamics, food pose and subject gaze
Source: Sci Data. 2022 Jan 12;9:5. doi: 10.1038/s41597-021-01101-8 (PMC8755801; doi:10.1038/s41597-021-01101-8)
Supplement: Supplementary file 1 — Supplementary Information [file 41597_2021_1101_MOESM1_ESM.pdf]

Flipping food during grilling tasks

A dataset of utensils kinematics and dynamics, food pose and subject gaze

Authors: **Débora Pereira *, Yuri De Pra, Emidio Tiberi, Vito Monaco, Paolo Dario, and Gastone Ciuti ***

* contacts: d.pereira@santannapisa.it; gastone.ciuti@santannapisa.it

Additional notes for the dataset users

Contents

Real inter-markers distance 3
Experimental conditions 3
Warning 4
Utensils dimensions 4
Markers label in the static trials with the food surfaces 5
Validation of the synchronization method used with S9 6

Real inter-markers distance

Table 1. Distance between pairs of markers placed on a same rigid segment, for the two utensils (spatula and tweezers).

utensil	utensil segment	markers pair	inter-markers distance (mm)
tweezers	cell_arm	c_long – c_far	75.5
		c_long – c_short	58.0
		c_short – c_far	61.8
	other_arm	a_long – a_far	74.2
		a_long – a_short	58.0
		a_short – a_far	51.0
	grasping_tip	c_tipother – c_tiplong	137.0
spatula	handle	hUpL – hUpR	118.1
		hUpL – hDownL	68.0
		hUpR – hDownR	65.0
		hUpL – hMid	50.0
		hUpR – hMid	68.4
		hDownR – hDownL	121.0
	blade	sUp – sR	62.0
		sUp – sClose	67.5
		sUp – sFar	64.5
		sR – sClose	71.5
		sR – sFar	73.0
		sClose – sFar	104.5

Experimental conditions

Table 2. Experimental conditions in which the subjects participated (in green) and did not participate (in red).

utensil	food	S1	S2	S3	S4	S5	S6	S7	S8	S9
spatula	fat hamburger	green	green	green	green	green	green	green	green	green
	light hamburger	green	green	green	green	green	green	green	green	green
	chicken breast	green	green	green	green	green	green	green	green	green
	zucchini middle, x-axis	green	green	green	green	green	green	green	green	green
	zucchini middle, y-axis	green	green	green	green	green	green	green	green	green
	zucchini tip, x-axis	green	green	green	green	green	green	green	green	green
	zucchini tip, y-axis	green	green	green	green	green	green	green	green	green
	eggplant	red	green	green	green	green	green	green	green	red
tweezers	zucchini middle, x-axis	green	green	green	green	green	green	green	red	green
	zucchini middle, y-axis	red	green	red	green	green	green	green	red	red
	zucchini tip, x-axis	green	green	green	green	green	green	green	green	green
	zucchini tip, y-axis	red	green	red	green	green	green	green	green	red
	eggplant	green	green	green	green	green	green	green	green	red

The type, and orientation (in case of zucchini slices) of the food flipped in each movement, is indicated in the list stored in the files “success_labels_S<subject_number>.csv”. This list follows the structure shown in Fig. 1.

	A	B	C	D	E	F	G
1	subject,foodtrial,foodtype,movementnumber,label,justification						
2	S1,hamb1,fat_hamburger,1,success,						
3	S1,hamb1,fat_hamburger,2,success,						
4	S1,hamb1,fat_hamburger,3,success,						
5	S1,hamb1,fat_hamburger,4,success,						
6	S1,hamb1,fat_hamburger,5,success,						
7	S1,hamb1,fat_hamburger,6,success,						
8	S1,hamb1,fat_hamburger,7,success,						
67	S1,chic2,chicken_breast,7,success,						
68	S1,chic2,chicken_breast,8,success,						
69	S1,chic2,chicken_breast,9,success,						
70	S1,chic2,chicken_breast,10,success,						
71	S1,chic2,chicken_breast,11,success,						
72	S1,chic2,chicken_breast,12,success,						
73	S1,zuch1,zucchini_middle_slice_X_axis,1,success,						
74	S1,zuch1,zucchini_middle_slice_X_axis,2,success,						
75	S1,zuch1,zucchini_middle_slice_X_axis,3,success,						
76	S1,zuch1,zucchini_middle_slice_X_axis,4,success,						
77	S1,zuch1,zucchini_middle_slice_X_axis,5,success,						
78	S1,zuch1,zucchini_middle_slice_X_axis,6,success,						
79	S1,zuch1,zucchini_middle_slice_X_axis,7,success,						
80	S1,zuch1,zucchini_middle_slice_X_axis,8,success,						

Figure 1. The file "success_labels_S<subject_number>.csv" contain a list of all the movements performed by "subject<number>". For each movement, there is the indication of: the trial ID, the type and orientation of the food, the movement number, the success/fail label, and the justification for that label.

Warning ⚠

All signals (motion, forces/torques, eye gaze, videos) were collected uniformly among subjects, including S9. However, as explained in the article, some technical difficulties in this day of experiments left available only one method to synchronize the signals: the detection of the precise instant in which the subject picked up the utensil from the table, which was a very evident event in the signals. So, the signals' synchronization method is the only difference in S9 with respect to the other subjects. Even if this difference is not expected to cause a significant bias in the data, users are advised to pay special attention to the data from these trials.

Utensils dimensions

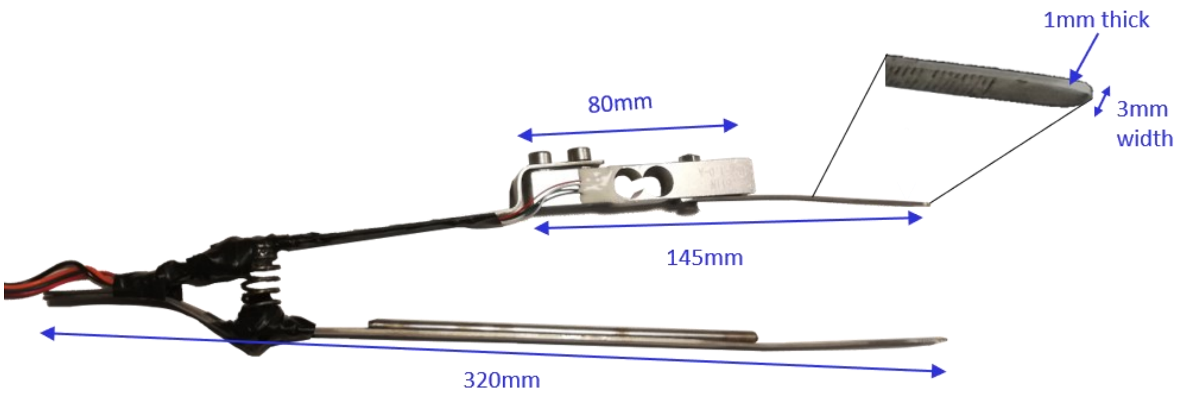

Figure 2. Dimensions of parts of the tweezers that were used in the experiments. One of the tips is zoomed in.

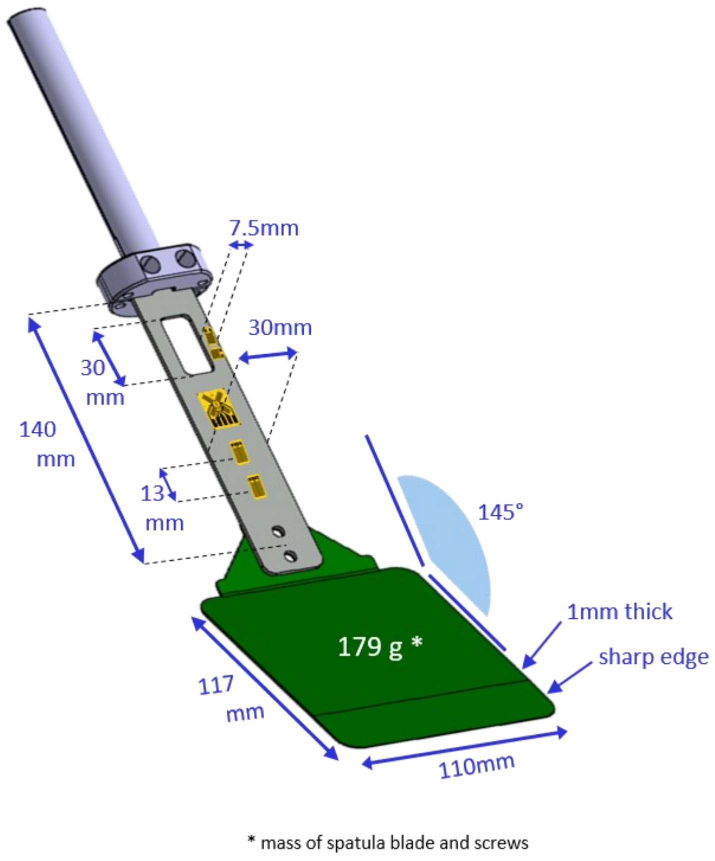

Figure 3. Dimensions and mass of parts of the spatula that was used in the experiments.

Markers label in the static trials with the food surfaces

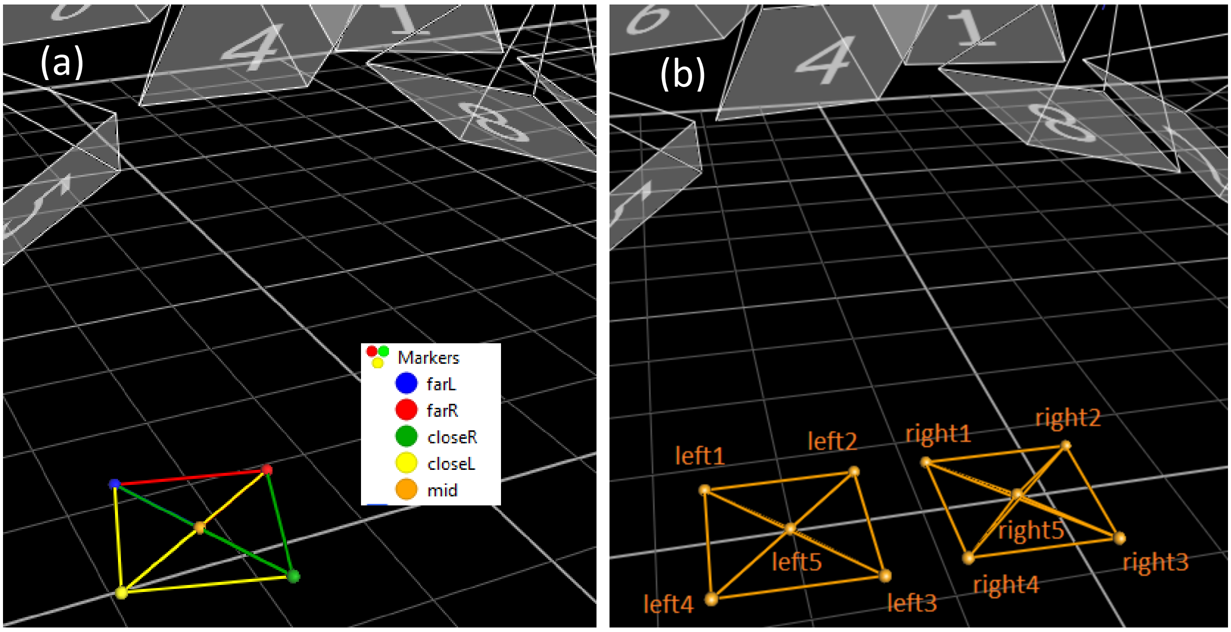

Figure 4. Names used, within the motion capture data, to label the markers placed on the surfaces of food. (a) Labels for the trials with subject S1; (b) Labels for the trials with the other subjects.

Validation of the synchronization method used with S9

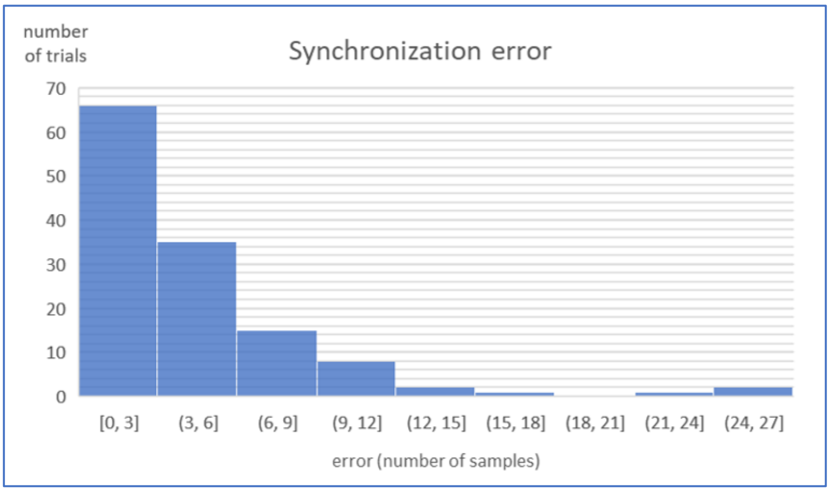

Distribution of the error obtained for the several trials of all the subjects except S9.
